# 3D Porous Zinc Scaffold Anodes for Enhanced Stability and Performance in Zinc-Ion Energy Storage Systems

**DOI:** 10.1021/acsnano.5c07729

**Published:** 2025-07-08

**Authors:** Xiaopeng Liu, Ruiqi Wu, Xueqing Hu, Alex M. Ganose, Jingli Luo, Iman Pinnock, Nibagani Naresh, Yijia Zhu, Yujia Fan, Tianlei Wang, Shuhui Li, Ivan P. Parkin, Buddha Deka Boruah

**Affiliations:** 1 Institute for Materials Discovery, University College London (UCL), London WC1E 7JE, U.K.; 2 Department of Chemistry, Imperial College London (ICL), London W120BZ, U.K.; 3 Department of Chemistry, University College London (UCL), London WC1H 0AJ, U.K.

**Keywords:** porous scaffold, 3D Zn, dendrite suppression, zinc anode, zinc-ion batteries/capacitors

## Abstract

Irregular Zn plating and stripping behaviors, along with the growth and detachment of Zn dendrites, pose a critical challenge to the rechargeability of zinc (Zn)-ion energy storage systems. In this study, a dynamic hydrogen bubble template (DHBT) method is introduced to construct an *in situ* 3D porous Zn scaffold on a Zn foil anode, which acts as a stable host to address morphological inhomogeneities during cycling. The pore walls provide abundant nucleation sites, effectively confining Zn growth within the scaffold and preventing vertical penetration into the separator. Consequently, the optimized 3D porous Zn scaffold symmetric cell exhibits a stable cycling life of over 1000 h at an areal current of 1 mA cm^–2^ and an areal capacity of 1 mAh cm^–2^. Furthermore, the modified 3D porous Zn scaffold anode delivers higher specific capacity and stability when paired with various cathode materials and electrolytes in full cell configurations, including Zn-ion batteries and Zn-ion capacitors. Significantly, the modified 3D porous Zn scaffold anodes demonstrate not only enhanced stability but also substantially improved charge storage performance compared to conventional Zn anodes, even under identical cathode conditions. This study underscores the critical role of surface modifications in Zn anodes, showcasing their ability to significantly enhance charge storage performance.

## Graphical abstract

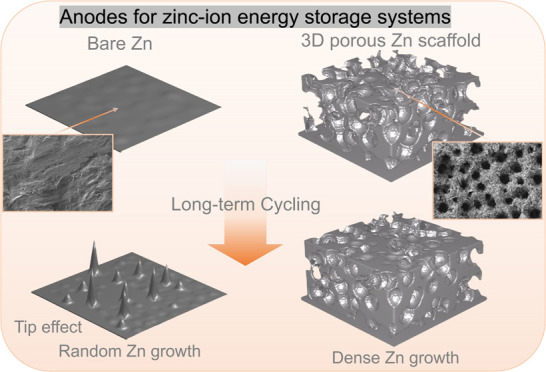

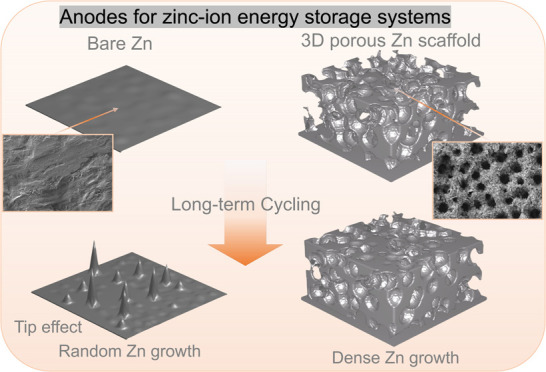

## Introduction

With advantageous features such as high safety, low cost, high theoretical capacity (820 mAh g^–1^), and low redox potential (−0.76 V vs SHE), zinc (Zn)-based energy storage systems, including batteries and capacitors, are considered promising candidates to meet the growing demand for next-generation minigrid and mini-off-grid energy storage applications.^1–3^ In these systems, Zn metal serves as the anode and is paired with various cathode materials, including battery-type materials for Zn-ion batteries or capacitor-type materials for Zn-ion capacitors.^4^ However, the commercial adoption of Zn-ion energy storage systems faces significant challenges, primarily stemming from uncontrolled dendrite growth on the Zn metal anodes.^5,6^ During cycling, the Zn stripping/plating process results in the continuous accumulation of Zn dendrites, leading to the formation of ″dead Zn″, which increases internal resistance and risks puncturing the separator, accelerating battery failure.^7,8^ Therefore, addressing these challenges through modifications of zinc metal anodes is essential.

Several strategies have been proposed to address these challenges, including solid electrolyte interphase (SEI) engineering, electrolyte and separator modification, structural design, and surface coating.^9–12^ For instance, Li et al. proposed a chelating-ligand additive strategy, which formed an inorganic/organic hybrid SEI bilayer interface to inhibit side reactions and regulated Zn-ion flux, thus improving the electrochemical performance of the Zn anode.^13^ Wang et al. constructed an antidendrite separator interlayer using a mass-producible hot-pressing strategy, which effectively promoted uniform nucleation and two-dimensional grain growth and improved the cycle stability.^14^ Among these, constructing 3D skeleton structures has proven to be an effective approach for mitigating dendrite formation by ensuring homogeneous current distribution, reducing the Zn^2+^ nucleation barrier, and providing uniform ion flux for Zn deposition.^15^ Carbon-based materials such as activated carbon,^16^ carbon nanofibers,^17^ and graphene^18^ have been explored as 3D carbonaceous scaffolds due to their high conductivity and low cost.^19^ For example, glucose-derived carbon has been used to construct a 3D continuous carbon network as a host for Zn deposition, offering more nucleation sites and mitigating dendrite formation, thereby extending cycling life.^20^ Additionally, certain 3D metal scaffolds, such as Cu^21^ and stainless steel,^22^ have demonstrated advantages for Zn deposition due to their structural stability and high electrical conductivity. However, the Zn affinity of the framework plays a complex role in determining the electrode performance. Scaffolds with weak Zn affinity often result in high overpotentials during Zn deposition, which can impair the cycling stability of the cell.^23^ On the other hand, if the Zn affinity of a deposition layer is too strong, then it can lead to preferential Zn^2+^ reduction on the deposition layer itself rather than on the anode surface, thereby compromising its protective function.^24^ Moreover, the significant weight of inactive materials in frameworks inevitably reduces the specific capacity of the batteries.^25,26^

To address these challenges, we employed the DHBT method to construct a 3D porous Zn scaffold on a Zn metal electrode in this study. This method utilized hydrogen bubbles as a dynamic template without requiring additional template additives. Furthermore, we explored the impact of the porous structure on the lifetime of batteries and capacitors, demonstrating through both simulations and experimental results that the structural configuration and Zn orientation play crucial roles in capacity decay. The interconnected pores of the proposed 3D porous Zn scaffold not only effectively suppressed uneven dendrite growth during the Zn plating/stripping process but also enhanced ion migration via ion diffusion channels. As a result, half cells featuring 3D porous Zn scaffold anodes exhibited a significantly extended lifespan of over 1000 h, compared to approximately 360 h for pristine Zn counterparts. When paired with various cathodes, such as PANI and V_2_O_5_ for battery applications and activated carbon for Zn-ion capacitors, these full cells demonstrated improved electrochemical performance using the 3D porous Zn scaffold anodes. We believe that our proposed design not only stabilizes the cycling performance of Zn metal anodes in Zn-based energy storage systems but also improves the charge storage performance, contributing to the realization of high-performance Zn-related energy storage systems.

## Results and Discussion

The effectiveness of Zn stripping and plating on Zn anodes is strongly influenced by their surface morphologies. Epitaxial growth behavior changes with variations in surface morphology, which ultimately determines the anode’s stability. To gain a comprehensive understanding of Zn stripping and plating on conventional Zn anodes and 3D porous Zn scaffolds (3D Zn), we studied their detailed performance. Figure 1a presents a schematic comparison of the cycled anodes in bare Zn and 3D Zn in full Zn-based energy storage cells. It is noted that bare Zn suffer from severe Zn dendrites after cycling due to the tip effects and random Zn growth that significantly shorten the lifespan of Zn-ion batteries during cycling (see further).^27,28^ In this work, a 3D porous Zn scaffold anode acts as the skeleton during the Zn plating/stripping process. It provides not only specific nucleation sites but also uniform Zn^2+^ flux, thereby guiding a smooth and homogeneous Zn plating behavior. The detailed analysis is shown later in this manuscript. As shown in Figure 1b, the structural evolution of the bare Zn and 3D Zn with different deposition times are illustrated by the intensity ratio of X-ray diffraction (XRD) patterns. The diffraction peaks around 36, 39, and 43° correspond to the (002), (100), and (101) crystal planes of pure Zn metal with a typical hexagonal close-packed (hcp) structure (Zn, PDF#04-0831).^29^ Notably, the intensity of the (002) peak gradually diminished with the 3D porous Zn scaffold deposition time continuing for 520 and 540 Zn. This phenomenon implies the unfavorable growth of the (002) plane in the porous Zn scaffold while preferential exposure of the (101) plane on its lateral sides (see further). Based on the earlier study, it is reported that the Zn deposition on the (101) textured zinc maintains a stable vertical epitaxial growth pattern with faster mass transfer kinetics, facilitating sustained and stable regulation, while (002) textured Zn can manage the planar growth of Zn flakes in the early stage; however, with the accumulation of lattice distortion, dendrites are eventually triggered.^30^ Therefore, we expect that the (101) textured rich 3D porous Zn scaffold could facilitate dense Zn growth compared to the bare Zn with random Zn growth with dendrites. Interestingly, the calculated intensity ratio of I_(101)_/I_(002)_ achieves 1.78 for the 540 electrode but drops to 1.16 for that of the 560 electrode (Table S1). To recognize the structural differences among the prepared 3D porous Zn scaffold samples, the top and cross-section SEM images of the prepared samples have been included (Figure 1c–j and Figure S1). The surface of bare Zn is relatively smooth with scattered grooves, which can easily lead to uneven growth of Zn dendrites. In contrast, the morphology of the as-prepared 3D porous Zn scaffold samples shows interconnected channels, around 5–10 μm in diameter, built on a flat Zn metal (Figure 1d,e,h,i). The hierarchical scaffold structure is beneficial to electrolyte permeability and ion transfer, indicating higher charge storage capability and cycling stability (discussed later). Although the uniformity of pore size could be maintained for 520 and 540 3D Zn, the porous channels in 560 3D Zn become indiscernible by massive Zn growth, which is probably due to the prolonged electrodeposition time (Figure 1f,j). These covered channels show a decrease in ordering and explain the I_(101)_/I_(002)_ drop for the 560 3D Zn. Figure S1 shows low-magnification SEM images and cross-sectional SEM views of the prepared samples. Compared with the thin deposition layer of the 520 3D Zn electrode around 17 μm, a denser layer around 27 μm thickness with a clearer porous configuration is shown for the 540 3D Zn electrode due to the longer treatment time. Instead, the configuration of the 560 3D Zn electrode is slightly covered by the overgrown Zn from the cross-section view, which agrees with the observations from Figure 1f,j. Figure 1k–n shows the contact angle of the 3D porous electrode toward the water droplet to associate the structure evolution with the hydrophilicity of the samples. The contact angles of 3D porous samples decrease from 67.66 to 56.85° for 520 Zn and 540 Zn, which are both smaller than that of the bare Zn (88.06°). The improved wettability between the porous structure and water droplet can be attributed to the increasing number of ion channels and porous surface, inducing better permeability of the aqueous electrolyte across the electrode and the homogeneous distribution of the Zn^2+^ flux. Based on the classical Wenzel theory, increased surface roughness amplifies surface wettability effects rather than a flat surface.^31^ In this regard, the increased roughness on the weakly hydrophilic surface (88.06° for bare Zn) is expected to achieve an enhanced wetting result in the contact angle measurement. Figure 1n shows that the contact angle of the 560 nm electrode recovered back to 74.12°, which is explained by the relatively smoother surface morphologies. The findings are consistent with XRD and SEM results. To understand the role of different surface orientations in crystal epitaxial growth, the adsorption energy of a Zn atom on different surface terminations was calculated using density functional theory (DFT). All symmetrically inequivalent single-atom adsorption sites were considered for both the (001) and (101) surfaces, with the lowest energy ground-state structures selected to determine the adsorption energy. As shown in Figure 1p, adsorption on the (101) plane exhibits a much lower energy value compared to that on the (001) plane. This suggests a higher stability and preferential epitaxial growth on the (101) surface, in good agreement with experimental observations.

**Figure 1:**
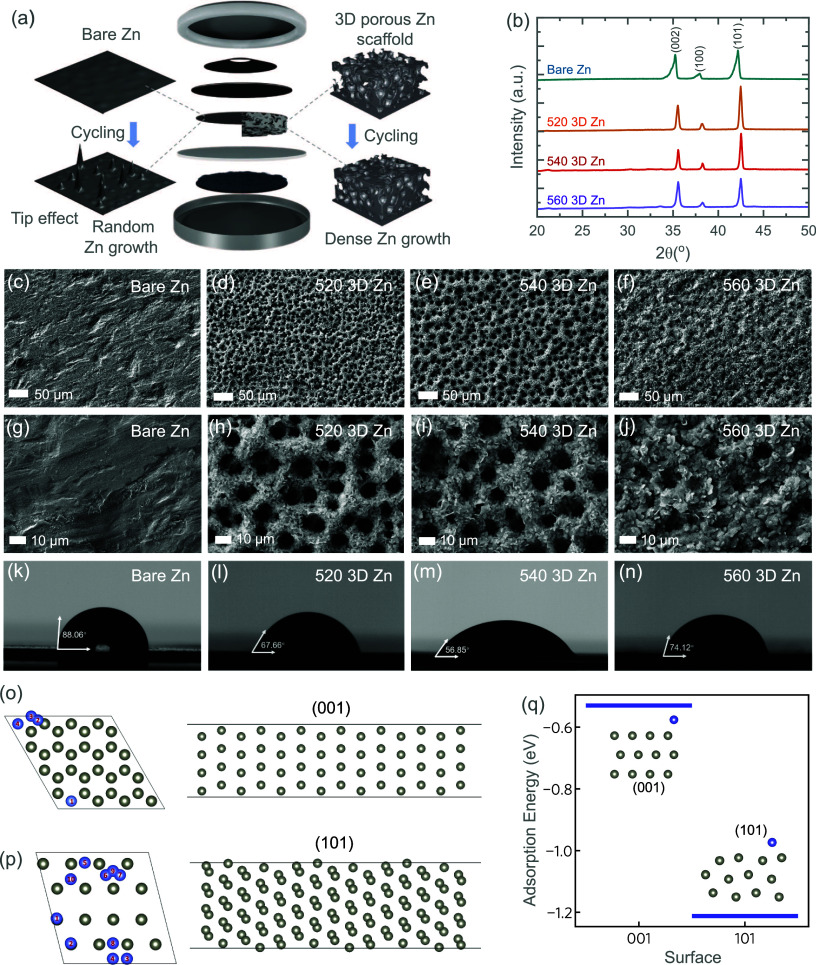
(a) Schematic illustration of Zn-based energy storage systems, comparing conventional Zn and 3D Zn anodes. After cycling, bare Zn anodes experience severe dendrite growth due to tip effects and random Zn deposition, whereas the porous Zn scaffold facilitates dense Zn growth through epitaxial deposition. (b) XRD patterns of 3D Zn and bare Zn anodes. Low- and high-magnification SEM images of (c, g) bare Zn and 3D Zn anodes at (d, h) 520, (e, i) 540, and (f, j) 560 conditions. Contact angles of water droplets on (k) bare Zn, and 3D Zn electrodes at (l) 520, (m) 540, and (n) 560 conditions. Crystal structure and Zn adsorption sites on the (001) and (101) surfaces (o, p). (q) Calculated Zn atom adsorption energy on different adsorption surfaces. Here, we illustrate only the energy of the most stable adsorption site.

The electrochemical behavior of 3D Zn electrodes is investigated by using symmetrical cells to evaluate the role of the 3D structure in regulating Zn stripping/plating in aqueous zinc-ion energy storage systems. Zn//Zn and 3D Zn///3D Zn symmetrical cells are cycled at an areal current of 1 mA cm^–2^ and an areal capacity of 1 mAh cm^–2^ (Figure 2a). Notably, the 540 3D Zn///540 3D Zn symmetric cell exhibits a low voltage hysteresis of 36 mV initially, which stabilizes at 30 mV after 1000 h. Enlarged views of Figure 2a highlight the exceptional stability of the 540 nm electrode during Zn plating/stripping. In contrast, the voltage profile of the Zn//Zn symmetric cell displays significant voltage fluctuations after 360 h, leading to a rapid short circuit. At a larger current density that is more likely to cause severe dendrite growth (5 mAh cm^–2^), the bare Zn electrodes in symmetric cells show voltage hysteresis escalation and rapid short circuit around 143 h, whereas the symmetrical cells based on a 540 3D Zn electrode manage to smoothly run for over 250 h (Figure S6). As shown in Figure S7, the voltage hysteresis of the 3D Zn//3D Zn symmetric cell decreases with continued deposition, indicating reduced concentration polarization due to enhanced ion diffusion at the electrode–electrolyte interface facilitated by the 3D porous structure. However, the cycling performance of 520 and 560 electrodes was inferior to that of the bare Zn symmetric cell. To further analyze the Zn deposition behavior, SEM images of cycled electrodes were captured at low and high magnifications (Figure 2b–e and Figure S8). The initially smooth surface of bare Zn became rough and exhibited severe Zn dendrite formation (Figure 2b,d), caused by charge accumulation around the initial Zn clusters. This uneven electric field, induced by large overpotential, led to irregular Zn deposition and eventual cell failure. In contrast, the 540 3D Zn electrode maintained a uniform surface without visible dendrite formation after 1000 h of cycling (Figure 2c,e). Zn growth progressively filled the channels rather than forming bulk deposits on the surface, confirming simulation results. The preferential exposure of the Zn (101) phase adjacent to the pore walls provided numerous nucleation sites, guiding epitaxial Zn growth and effectively covering the pores over time. As shown in Figure S8a,b, although the original pore morphology of the 520 electrode is retained after cycling, there are some collapses in the 3D deposition layer. This likely occurs because the thin 520 electrode, with insufficient Zn nucleation sites, cannot effectively sustain prolonged Zn growth within the host material.^32^ The limited number of nucleation sites generates significant stress on the structure, eventually leading to its failure. In contrast, the porous channels of the 560 electrode become covered with Zn dendrites, which significantly shortens the cell lifespan (Figure S8c,d). A detailed analysis of this failure will be presented later in the manuscript. Figure 2f compares the plating/stripping profiles of Zn//Zn and 3D Zn//3D Zn symmetric cells across various areal currents, ranging from 0.1 to 5 mA cm^–2^. The bare Zn cell clearly struggles to maintain low voltage hysteresis compared to that of the 540 electrodes. These findings highlight that the interconnected porous channels in the prepared 3D Zn electrodes enhance ion diffusion and lower energy barriers for Zn nucleation, resulting in more stable and controlled Zn plating/stripping behavior. This conclusion is further supported by electrochemical impedance spectroscopy (EIS) measurements of symmetric cells at different temperatures (Figure 2g,h and Figure S9). In the EIS profiles, the semicircles observed in the high-frequency region correspond to the charge transfer resistance (*R*_ct_) of the electrodes. The *R*_ct_ of the symmetric cell using bare Zn (Figure 2g) is nearly five times higher than that of the symmetric cell with 540 3D Zn (Figure 2h) at room temperature, demonstrating the better ionic diffusion efficiency of the 540 3D Zn electrode. To further evaluate the Zn^2+^ diffusion kinetics, the activation energy (*E*_a_) was calculated using the Arrhenius equation:^33^

$$\frac{1}{R_{\mathrm{ct}}}=A\exp(-E_{a}/RT)$$

where *A*_0_ represents a pre-exponential constant, *E*_a_ is the activation energy, *k* is a gas constant, and *T* is the absolute temperature. Compared to the bare Zn with the largest calculated *E*_a_ value of about 50.3 kJ mol^–1^, the *E*_a_ value gradually drops to 33.5 and 28.9 kJ mol^–1^ for 540 3D Zn and 560 3D Zn, respectively (Figure 2i and Figure S10). The trend can be well supported by the above long-term cycling and rate performance profiles. This improvement in interfacial kinetics confirms that the construction of the 3D scaffold effectively aids in desolvating the Zn^2+^ sheath at the electrode–electrolyte interface, thereby promoting rapid ion transfer and homogenizing Zn-ion deposition. Moreover, the comparative Tafel plot is obtained in 2 M ZnSO_4_ electrolyte to further explore the interfacial thermodynamic behavior of the electrodes (Figure 2j). In contrast to the high corrosion current of 8.01 mA cm^–2^ for the bare Zn electrode, the 540 3D Zn electrode exhibits a lower value of 5.49 mA cm^–2^, indicating a retardant corrosion activity. Similarly, the comparative LSV curve demonstrates that the potential is extended from −1.008 V (bare Zn) to −1.038 V (540 3D Zn) at a given current (−10 mA cm^–2^), reflecting the reduced tendency of the porous electrode toward the hydrogen evolution reaction (Figure 2k). To confirm the Zn deposition behavior of the electrodes, chronoamperometry (CA) tests are conducted in a symmetric cell under a constant overpotential of −150 mV for 600 s (Figure 2l). The areal current of bare Zn presents a continuous change throughout the measured time, indicating an undesired 2D diffusion process of Zn^2+^ that results in Zn aggregation and dendrite growth. In contrast, the areal current of 540 3D Zn becomes stable after 80 s, reflecting a 3D diffusion process that facilitates smooth Zn deposition behavior. Zn^2+^ transference number (*t*_Zn_) is a critical indicator of Zn^2+^ migration kinetics at the anode–electrolyte interface. The faster migration behavior would relieve concentration polarization and hinder Zn dendrite growth. *t*_Zn_ could be calculated by the equation below:^34^

$$t_{\mathrm{Zn}}=\frac{I_{s}(\Delta V-I_{0}R_{0})}{I_{0}(\Delta V-I_{s}R_{s})}$$

where Δ*V* (10 mV) represents the applied voltage, *I*_0_ is the initial current, *I*_s_ is the steady-state current, and *R*_0_ and *R*_s_ denote the electrode interface impedance before and after the polarization, respectively. As shown in Figure S11, *t*_Zn_ increases from 0.21 to 0.47 after introducing a 3D porous design. The accelerated Zn^2+^ kinetics implies more uniform Zn-ion flux and further verifies the smooth Zn electrodeposition behavior on the 3D Zn surface.

**Figure 2:**
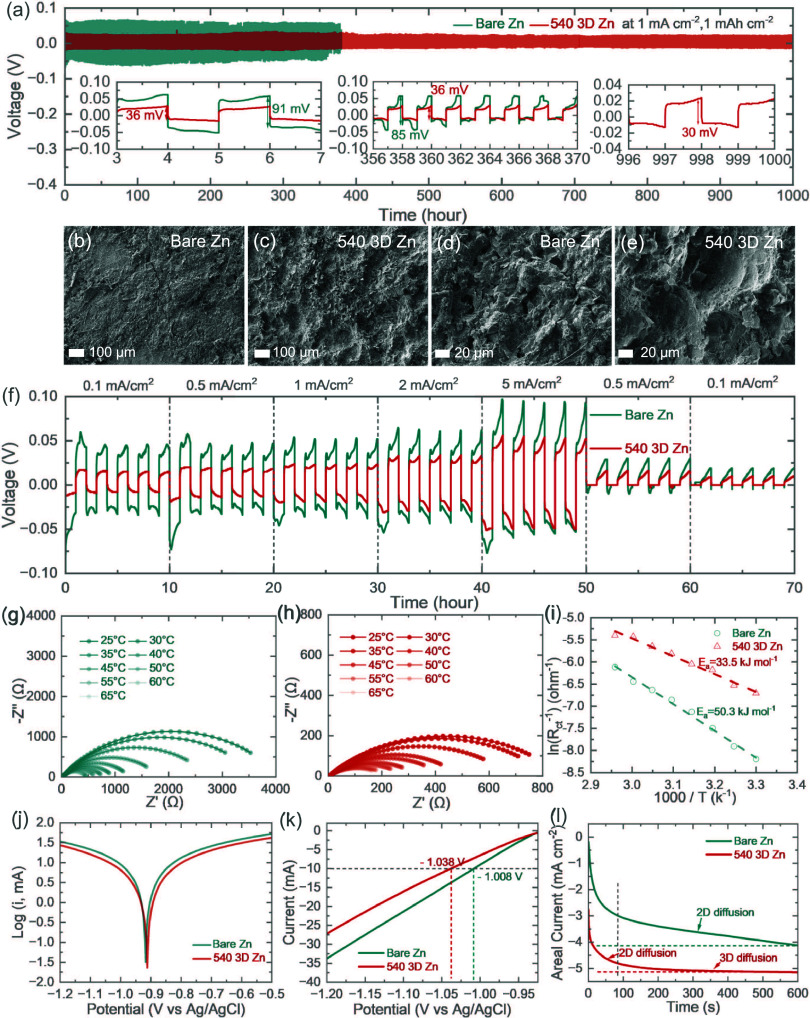
(a) Long-term cycling tests of the symmetric cells at an areal current of 1 mA cm^–2^. Post-SEM images of the cycled (b) bare Zn and (c) 540 3D Zn electrodes at low magnifications and the same (d) bare Zn and (e) 540 3D Zn electrodes at high magnifications. (f) Rate performance of the symmetric cells. Nyquist plots of the symmetric cells based on (g) bare Zn and (h) 540 3D Zn at different temperatures. (i) Comparative activation energy plot for bare Zn and 540 3D Zn anodes. (j) Tafel plots, (k) LSV curves, and (l) CA tests of bare Zn electrodes and 540 3D Zn electrodes.

Figure 3a,b and Figure S12 present the *in situ* optical microscopy to observe the impact of our structure design in the Zn dendrite growth at a high areal current of 10 mA cm^–2^. In the 3 M Zn­(CF_3_SO_3_)_2_ electrolyte, all of the samples exhibit a smooth surface at the initial stage. After 15 min, moss-like Zn starts to emerge on the bare Zn (Figure 3a­(ii)). The Zn dendrite continues to extend alongside the aggregates progressively over time (Figure 3a­(iii)) and eventually cover the surface of the bare Zn (Figure 3a­(iv)). On the contrary, the surface on 540 3D Zn samples remains uniform and smooth, with no noticeable Zn dendrite observed within 60 min (Figure 3b). The surface change of the electrodes after 60 min cycling are further observed by SEM images. Figure 3c,d depicts the dendritic status of the bare Zn, where the large protruding particles aggregated on the surface. The cross section of the corresponding electrodes is further examined (Figure 3e). As expected, the bare Zn experiences obvious dendrite growth, resulting in a deposition layer that is around 100 μm thick. In contrast, with the introduction of a 3D scaffold, Zn deposits in a regular and controllable manner that inhibits the growth of Zn dendrite. As evident from Figures S14a and S15a,b, the surface of 520 3D Zn remains relatively flat, and the Zn dendrite is grown within the structure that seals the holes rather than accumulate on the surface. Moreover, the dendrite deposition layer of 520 3D Zn is around 10 μm thick (Figure S15c), which is much smaller than that of bare Zn. Remarkably, the porous structure remains clear and dendrite-free for 540 3D Zn (Figure 3f,g). Figure 3h and Figure S13 display a uniform Zn deposition behavior of 540 3D Zn with no visible Zn dendrite from the cross-section views. The completed 3D structure of 540 3D Zn provides numerous nucleation sites to promote uniform Zn deposition. In contrast, Figure S15d,e show that Zn prefers to grow planarly on the surface of 560 3D Zn molecules rather than fill the holes, hiding them in the dark region. Figure S15f shows a clear surface of 560 3D Zn after cycling from a cross-section view, which is consistent with the observation from Figure S14b. The directions of Zn deposition on the samples are consistent with the simulation and XRD results above. To get a deep insight into the effect of Zn plating/stripping behavior on the samples for a long period, the symmetric cells with the as-prepared electrodes are cycled in a transparent cuvette without a separator for 5 days. The cuvettes are performed at an areal current of 1 mA cm^–2^, and the corresponding *in situ* digital images are provided in Figure 3i,k and Figure S16. All the sample surfaces are smooth and clear at the beginning (day 0). As illustrated in Figure 3i­(ii), uneven Zn^2+^ nucleation on the bare Zn is evident after 24 h. This issue becomes increasingly pronounced over time (Figure 3i­(iii)), which results in large protruding dendrites observed on the surface after deposition for 5 days (Figure 3i­(iv)). Although less dendrite growth is shown on 520 3D Zn, sharp protrusions capable of penetrating the separator are observed owing to the “tip effect”, indicating insufficient inhibition effect of the dendrite formation (Figure S16a). In contrast, Zn deposition on the surface of 540 3D Zn is homogeneous with no noticeable Zn dendrites formed throughout the tested time (Figure 3k). As for 560 3D Zn, the deposition layer is peeled off from the surface and fails to endure the volume change during the repeated Zn plating-stripping process (Figure S16b). The cycled electrodes are taken out and dried for SEM after 5 days to further examine their morphology change. In contrast to the cracked dendritic layers on the surface of the bare Zn (Figure 3j and Figure S18a,b), the deposited surface of the 540 electrode remains smooth (Figure 3l). Furthermore, vertically aligned Zn is found on the 3D skeleton (light region), indicating the epitaxial Zn growth by the guidance of the Zn (101) phase within the structure (Figure S18f). The corresponding cross-sectional SEM images reveal rampant dendrite growth on the bare Zn anode after deposition, whereas the 3D coating suppresses dendrite formation, preserving surface flatness (Figure S17). Figure S18c shows the even surface of 520 3D Zn, indicating the successful suppression of the Zn dendrite even with the less dense porous structure. The formation of scattered protrusions may be attributed to the insufficient nucleation sites on the Zn channels due to the short electrodeposition time (Figure S18d). The Zn growth at these limited sites may create localized charge accumulation across the surface and encourage the formation of protrusions overtime. The detached layer of the 560 electrode is examined to explore the potential reason for the failure of the symmetric cell in the long-term cycling test (Figure S18g,h). Generally, the energy release rate (*G*) of different deposition layers, a key driver for crack formation, is compared by the equation below:^35^

$$G\propto \frac{\sigma^{2}h}{E}$$

where σ is the in-plane tensile stress, *h* is the film thickness, and *E* is the elastic modulus. Cracks start to form when the energy release rate surpasses the material’s fracture toughness. Based on this equation, the detachment is more likely to happen for 560 3D Zn due to the larger thickness than that of the others. In addition, consider the compact space within the channels of the 560 electrode (Figure 1f), Zn dendrites may exert larger pressure on the porous structure during the cycling process, potentially leading to the delamination when the pressure within the layer is higher that the critical value (Figure S18g,h). These observations clearly reaffirmed that the 540 electrode was the optimized choice to effectively guide uniform Zn deposition and explain their different performances during the electrochemical tests above.

**Figure 3:**
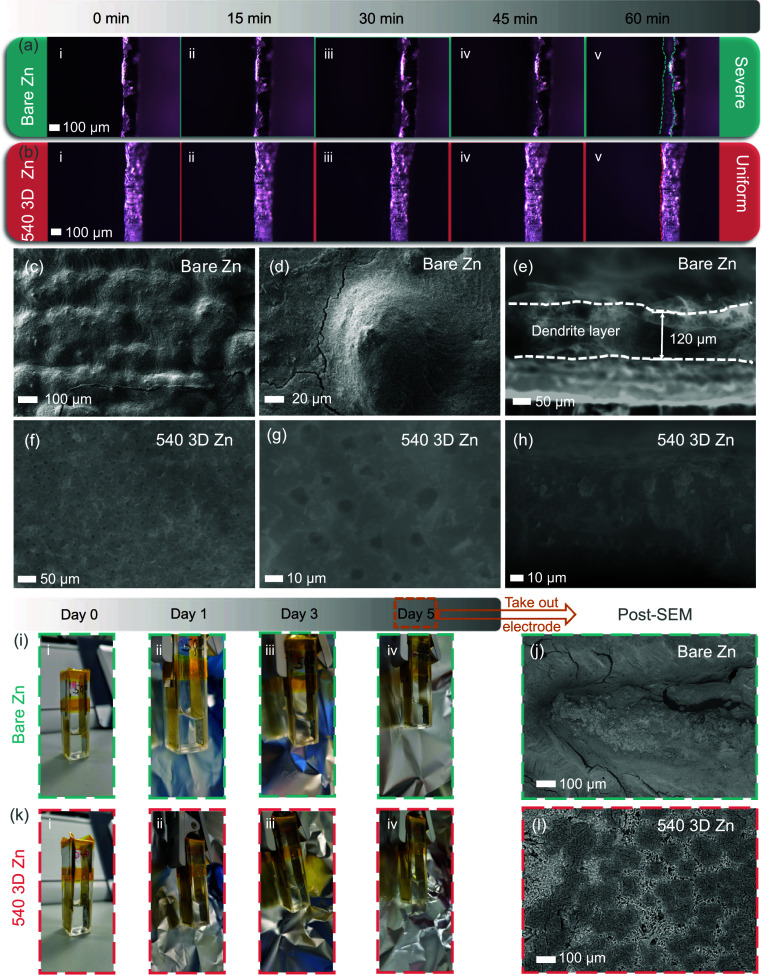
In-situ optical images of the Zn deposition process for (a) bare Zn and (b) 540 3D Zn captured at various time intervals. Postdeposition SEM images of bare Zn: (c, d) top view and (e) cross-sectional view after 60 min of Zn^2+^ plating. Postdeposition SEM images of 540 3D Zn: (f, g) top view and (h) cross-sectional view after 60 min of Zn^2+^ plating. In-situ digital images of the 1 × 1 cm^2^ cuvette cell were based on (i) bare Zn with corresponding post-SEM image (j) and (k) 540 3D Zn with corresponding post-SEM image (l).

To investigate the practical application of our designed 3D Zn, PANI, V_2_O_5_, and activated carbon (AC) cathodes are paired with our 3D Zn anodes for different electrochemical tests. Figures S19 and S20 present the related characterizations of the cathodes. The SEM images of the cathodes are shown at different magnifications in Figure S19, revealing the highly polarized fiber network of PANI (Figure S19a), the flake-like particles constructed by the hierarchical V_2_O_5_ nanowires (Figure S19b), and roughly stacked layers of AC powders (Figure S19c). Figure S20 reveals the Raman spectra of PANI, V_2_O_5_, and AC. Peaks at 1249, 1340, 1403, 1488, 1565, 1603, and 1640 cm^–1^ indicate the vibrational modes of PANI (Figure S20a), peaks at 139, 197, 288, 404, 528, 700, and 998 cm^–1^ confirm the vibrational modes of V_2_O_5_ (Figure S20b), and the two sharp peaks around 1350 and 1590 cm^–1^ correspond to the typical D and G bands of AC materials (Figure S20c).^36–39^ First, different prepared Zn anodes are paired with the PANI cathode within the voltage window of 0.5–1.5 V for full cell tests. Cyclic voltammetry (CV) measurements are conducted at different scan rates from 0.3 to 1.5 mV s^–1^ to further examine the charge storage behavior of the 3D Zn electrode and its pristine counterpart (Figure 4a,b). The energy storage process is driven by the interaction of conjugated (C=N) bonds, enabling anion association with oxidized PANI (C–N^+^), while cations are accommodated at reduced PANI (C–N^–^) sites. In this particular system, the triflate anion (CF_3_SO_3–_) associates with oxidized PANI during charging, whereas Zn^2+^ ions coordinate with reduced PANI during discharge. The positions of redox peaks align with the findings from previous studies.^40^ Here, diffusion-controlled and capacitive-controlled processes are quantified to understand the charge storage kinetics of the as-prepared electrodes (Figure 4c,d). The relationship between the measured peak (*i*) and the scan rate (*v*) follows the equation below:^41^

$$i=av^{b}$$

where *a* and *b* are variable parameters. In principle, *b*-value ≈ 1 indicates that the capacitive-controlled mechanism is governed; *b*-value ≈ 0.5 indicates that the diffusion-controlled mechanism is governed. The calculated *b*-values of peak 1, peak 2, peak 3, and peak 4 were 0.78, 0.958, 0.76, and 0.70 and 0.84, 0.989, 0.80, and 0.85 for the bare Zn and 540 3D Zn electrodes, respectively. This implies that the capacitive-controlled kinetics is favored by 540 3D Zn electrodes. Moreover, the capacity contributions are quantitatively divided into the capacitive-controlled (*k*_1_ *v*) and diffusion-controlled (*k*_2_ *v* ^1/2^) components by the equation below:^42^

$$i\left(V\right)=k_{1}v+k_{2}v^{1/2}$$

where *i* is the current response, *v* is the scan rate, and *k*_1_ and *k*_2_ are defined parameters. As shown in Figure 4f, the capacitive contribution of the 540 electrodes gradually increases with the scan rates, reaching 93.56, 94.63, 95.99, and 96.93% at scan rates of 0.6, 0.9, 1.2, and 1.5 mV s^–1^, respectively. The values surpass those for the bare Zn under identical conditions (Figure 4e), suggesting enhanced high-rate capability of the cells.

**Figure 4:**
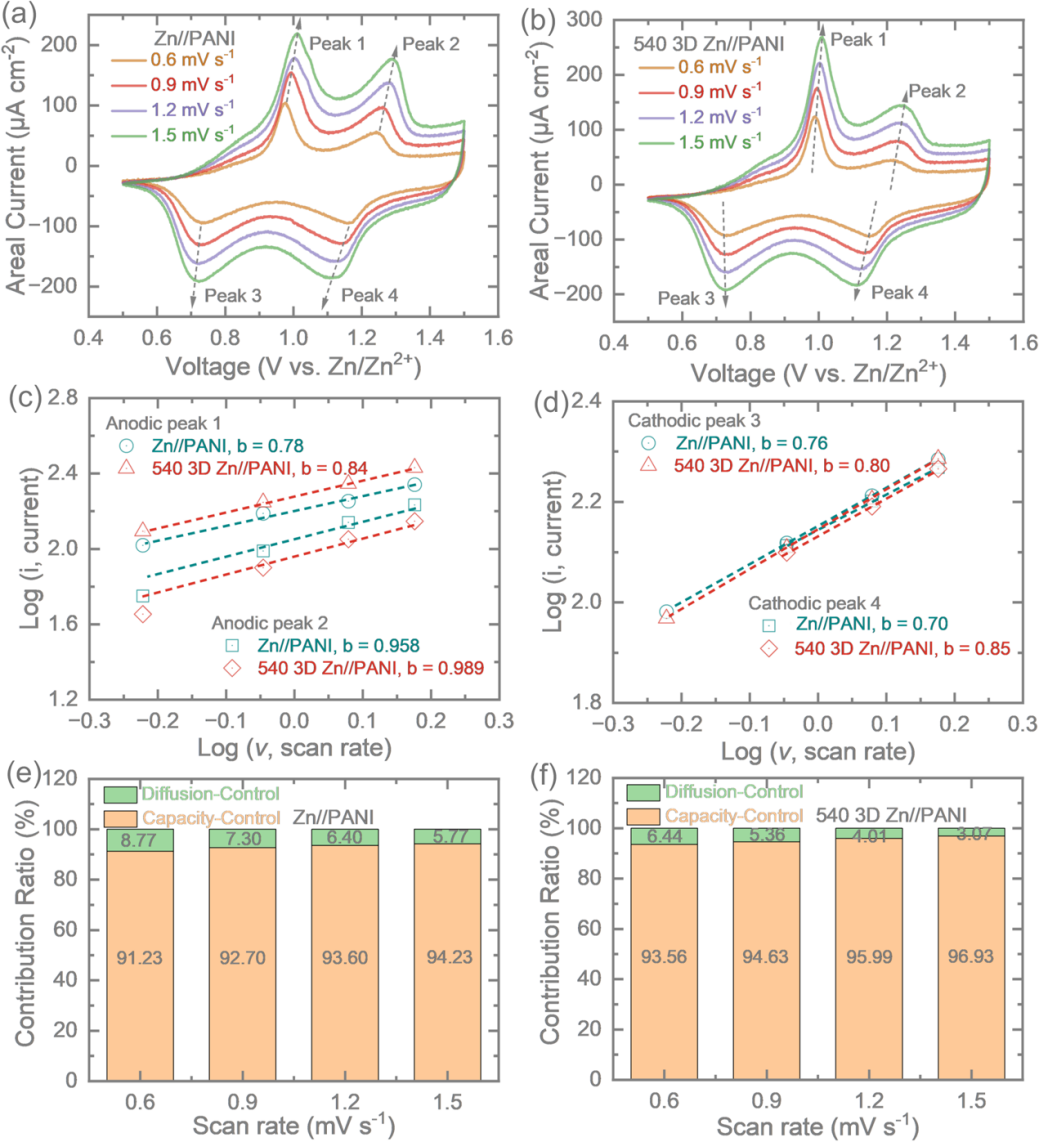
CV curves of (a) Zn//PANI and (b) 540 3D Zn//PANI. The calculated *b* value for the (c) anodic and (d) cathodic peaks of Zn//PANI and 540 3D Zn//PANI. Capacitive contribution ratio with respect to the scan rate of (e) Zn//PANI and (f) 540 3D Zn//PANI.

Our 3D Zn design is also coupled with an AC cathode to investigate its potential for a Zn-ion capacitor (ZIC) within the voltage window of 0.2 to 1.8 V using 2032 type coin cells. The CV curves of 540 3D Zn//AC ZIC deliver similar quasi-rectangular shapes as those of Zn//AC ZIC at multiscan rates, ranging from 2 to 50 mV s^–1^, confirming the stability of the structure (Figures S21a,b). Notably, the 540 mm 3D Zn//AC ZIC displays a 28.2% enhancement in the enclosed CV area compared to that of the Zn//AC ZIC at the scan rate of 5 mV s^–1^, which could be ascribed to the larger surface area of the 3D Zn surface and unimpeded ion migration at the interface (Figure S21c). The improved charge storage performance could also be maintained at a high scan rate of 50 mV s^–1^, revealing its potential at high-rate conditions (Figure S21d).

Figure 5a and Figure S22a display the galvanostatic discharge–charge (GDC) profiles of 540 3D Zn//PANI and Zn//PANI full cells at different specific currents between 100 to 2,000 mA g^–1^, with a voltage window between 0.5 and 1.5 V. The 540 3D Zn//PANI shows lower voltage hysteresis compared to that of Zn//PANI, providing solid evidence for the faster kinetics of the 540 3D Zn electrode discussed in the CV results and the tests for the symmetric cells (Figure 2f). Specifically, the midvalue potential of the discharge process at 100 mA g^–1^ is 1.04 V for 540 3D Zn//PANI, which is higher than that of Zn//PANI (0.94 V). During the charging process, this value becomes 1.14 and 1.10 V for 540 3D Zn//PANI and Zn//PANI, respectively (Figure 5b). When the specific current increases to 2000 mA g^–1^, the difference of midvalue voltage of 540 3D Zn//PANI is still 65 mV lower than that of Zn//PANI (Figure S22b), implying the enhanced electrode kinetics of 540 3D Zn. As shown in Figure 5c, the 3D Zn//PANI full cell exhibits higher specific capacities compared to its bare Zn counterpart at all of the measured specific currents. The specific capacities are kept at 142.85, 131.86, 121.37, 113.5, and 106.39 mAh g^–1^ for 540 3D Zn//PANI with an incremental increase in specific current, outperforming those of Zn//PANI (109.53, 103.84, 97.52, 92.17, and 85.89 mAh g^–1^, respectively). The significant enhancement observed, after introducing the 3D scaffold, may be caused by the improved Zn^2+^ plating/stripping kinetics that facilitate charge storage. The GCD curves of ZICs with different anodes are also investigated at various specific currents (Figure 5d,e and Figure S23). Based on the symmetric charge and discharge profiles, 540 3D Zn//AC ZIC exhibits reversibility during cycling (Figure 5d). It shows a specific capacity of 48.56 mAh g^–1^ at 200 mAh g^–1^ and maintained 24.32 mAh g^–1^ at 2000 mA g^–1^. Apart from that, 540 3D Zn possesses higher discharge capacities than that of bare Zn at all the measured specific currents, which could be attributed to the large surface area and abundant reactive sites of the 3D Zn scaffold as well as rapid ion migration at the interface (Figure 5f). In addition, our prepared 540 3D Zn//PANI achieves an energy density of 144.44 Wh kg^–1^ at a power density of 99.81 W kg^–1^ and retains 100 Wh kg^–1^ at 1884.82 W kg^–1^, and our 540 3D Zn//AC capacitor delivers an energy density of 16.22 Wh kg^–1^ at a power density of 701.53 W kg^–1^. Both devices show improved electrochemical performance compared to most of the Zn-ion batteries and ZICs reported in the literature, respectively (Figure 5g,h).

**Figure 5:**
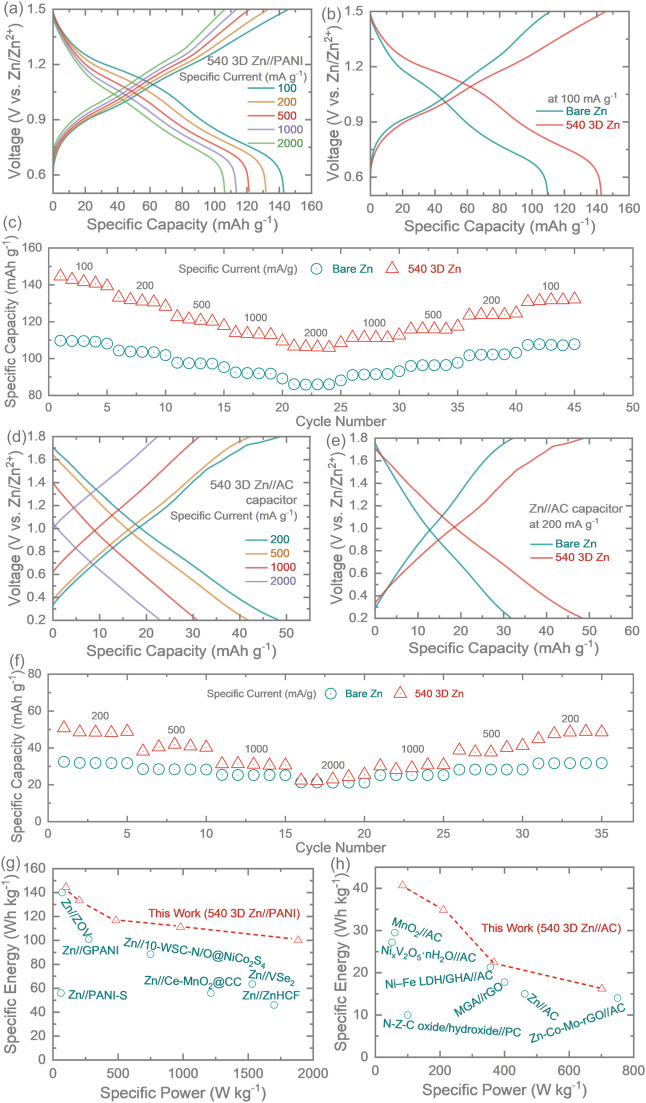
(a) GDC curves of a 540 mA 3D Zn//PANI full cell at different specific currents. (b) Comparative GCDs of Zn//PANI and 540 mA 3D Zn//PANI at specific currents of 100 mA g^–1^. (c) Rate tests of the Zn//PANI and 540 3D Zn//PANI full cells. (d) GCD curves of the 540 3D Zn//AC capacitor at different specific currents. (e) Comparative GCDs of Zn//AC and 540 3D Zn//AC at specific currents of 200 mA g^–1^. (f) Rate tests of the Zn//AC and 540 3D Zn//AC capacitors. (g) Ragone plot of our 540 3D Zn//PANI with other Zn-ion batteries reported in the literature: Zn//ZOV,^43^ Zn//GPANI,^44^ Zn//PANI-S,^45^ Zn//10-WSC-N/O@NiCo_2_S_4_,^46^ Zn//Ce-MnO_2_@CC,^47^ Zn//VSe_2_,^48^ and Zn//ZnHCF.^49^ (h) Ragone plot of our 540° 3D Zn//AC capacitor with other asymmetric capacitors reported in the literature: Zn–Co–Mo-rGO//AC,^50^ Zn//AC,^51^ MGA//rGO,^52^ N–Z–C oxide/hydroxide//PC,^53^ Ni–Fe LDH/GHA//AC,^54^ Ni_*x*_V_2_O_5_·nH_2_O//AC,^55^ and MnO_2_//AC.^56^

Figure 6a compares the long-term cycling performance of 540 3D Zn//PANI and Zn//PANI at a specific current of 1000 mA g^–1^. Different from the stable cycling of 540 3D Zn//PANI with a specific discharge capacity of 169.6 mAh g^–1^ cm^–2^ (capacity retention ∼98.22%) after 300 cycles, the Zn//PANI full cell delivers an initial specific capacity of 120.32 mAh g^–1^ and then rapidly decays to 81.79 mAh g^–1^ (capacity retention ∼62.29%) within the same period of time. Figures S24 and S25 include the related characterizations for the cycled PANI electrodes to remove the concern about the effect of PANI on the cell failure. Figure S24 shows the Raman spectra of PANI cathodes in Zn//PANI and 540 3D Zn//PANI after cycling. The peaks corresponding to PANI, indicated by the red dotted lines, are consistent with a previous report.^37^ The peak shown by the blue dotted line belongs to the D bands of graphene, while the peak of G bands at 1572 cm^–1^ is merged by the peak of PANI. Figure S25 compares the morphology of the PANI cathodes after cycling. The surface of PANI cathodes from Zn//PANI is almost similar in morphology to that of 540 3D Zn//PANI, suggesting that the difference in battery stability is not primarily caused by the PANI cathode. Figure S26 reveals a sharp contrast between the surfaces of the two samples after cycling. The bare Zn is covered by moss-like dendrite and scattered protrusions, while the surface of 3D Zn retained the dendrite-free status with an intact porous structure. The improved stability of the 540 electrode could be further validated by the comparison of the EIS results before and after cycling (Figures S27 and S28 and Table S3). The *R*_ct_ of Zn//PANI rapidly increases from 1309 to 2405 Ω after cycling, probably due to the formation of byproducts shown in the post-SEM images. In contrast, the *R*_ct_ of the 3D Zn//PANI shows a smaller value of 272 Ω at the beginning and is maintained relatively stable after cycling (389 Ω). The reduced impedance of 3D Zn could be attributed to efficient Zn-ion diffusion and inhibition of Zn dendrite growth, which improves stability and agrees with the SEM and long-term cycling results. Furthermore, both 3D Zn and bare Zn are paired with V_2_O_5_ electrodes (Figure 6b) and AC (Figure 6c) in 3 M Zn­(CF_3_SO_3_)_2_ and 1 M Zn­(CF_3_SO_3_)_2_ aqueous electrolytes, respectively. Similarly, 3D Zn showed better stability in these applications; the full cell based on 540 3D Zn and V_2_O_5_ electrodes shows an enhanced specific capacity of 70.77 mAh g^–1^ (capacity retention of 70.57%) at a current density of 1000 mA g^–1^ after 900 cycles, which is two times higher than that of Zn//V_2_O_5_ (34.85 mAh g^–1^, capacity retention of 47.64%). Similarly, AC paired with the 3D Zn anode (540 3D Zn//AC) demonstrates improved stability compared to the pristine Zn anode (AC//Zn) (Figure 6c). The 540 3D Zn//AC capacitor shows good capacity retention of 111.24% after 10,000 cycles, which is much higher than that of the Zn//AC capacitor (54.84% after 10,000 cycles). This discrepancy in cycling stability between the two anodes could be contributed to the unsuppressed side reactions and uneven Zn deposition behavior of the bare Zn, evidenced by the significantly fluctuated Coulombic efficiency of the Zn//AC capacitor after 5500 cycles.

**Figure 6:**
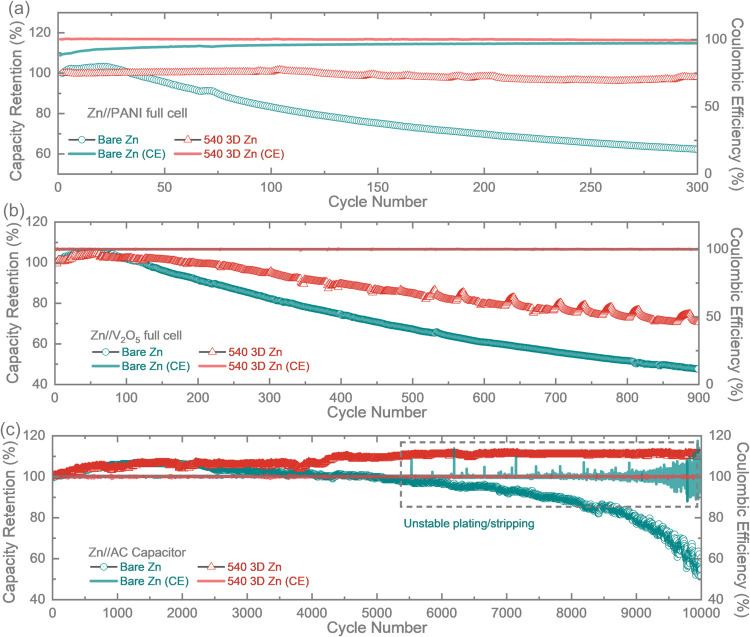
(a) Long-term cycling performance of Zn//PANI and 540 3D Zn//PANI at a specific current of 1000 mA g^–1^. (b) Long-term cycling performance of Zn//V_2_O_5_ and 540 3D Zn// V_2_O_5_ at a specific current of 1000 mA g^–1^. (c) Long-term cycling performance of ZICs at a specific current of 5000 mA g^–1^.

## Conclusions

In conclusion, a mesoporous 3D hierarchical network was successfully constructed on the Zn anode surface by using a dynamic hydrogen bubble template (DHBT) method for Zn-ion storage applications. Simulations and experimental characterizations reveal that the epitaxial effect of the Zn (101) planes promotes Zn-ion deposition along the lateral pore walls of the 3D scaffold, encapsulating Zn growth within the host structure and preventing vertical penetration into the separator. By leveraging reduced nucleation overpotential and epitaxial Zn growth, the optimized 540 3D Zn electrode achieves exceptional cycling stability, maintaining a smooth surface over 1000 h at an areal current of 1 mA cm^–2^. Notably, the 540 3D Zn//PANI full cell exhibits a high specific capacity of 169.6 mAh g^–1^ with an impressive capacity retention of 98.22% after 300 cycles, significantly outperforming the bare Zn counterpart (81.79 mAh g^–1^ with 62.29% retention). Additionally, the enhanced electrochemical performance of the 3D Zn electrode was validated in other Zn-based systems, including Zn//V_2_O_5_ batteries and Zn//AC capacitors. This work offers valuable insights into the rational design of 3D Zn anodes, paving the way for the development of highly stable and efficient Zn-based energy storage systems.

## Experimental Section

### Chemical Reagents

All of the reagents were directly used as received. Sodium bromide (NaBr) was purchased from Fluorochem. Aniline, sulfuric acid (H_2_SO_4_), zinc acetate dihydrate (C_4_H_6_O_4_Zn·2H_2_O), vanadium­(V) oxide (V_2_O_5_), polyvinylidene fluoride (PVDF), and *N*-methyl-2-pyrrolidone (NMP) were purchased from Sigma-Aldrich. Ammonium acetate (C_2_H_3_O_2_NH_4_) was obtained from Source Chemicals. Hydrogen peroxide (H_2_O_2_, 30% w/v) was acquired from Fisher Chemical. Activated carbon (AC) paste was purchased from DEP Technologies. Graphene paper was purchased from Ossila. Carbon graphite paper was acquired from SGL Carbon. Metallic zinc foil was supplied by Goodfellow. For electrolyte preparation, zinc trifluoromethanesulfonate (Zn­(CF_3_SO_3_)_2_) and guar gum was purchased from Sigma-Aldrich.

### Electrodeposition of the 3D Porous Zn Electrode

The 3D porous Zn electrode was deposited on the flat Zn foil by a dynamic bubble strategy. First, the electrodeposition electrolyte containing 15.434 g of NaBr, 0.109 g of C_4_H_6_O_4_Zn·2H_2_O, and 3.854 g of C_2_H_3_O_2_NH_4_ was mixed in 50 mL of DI water. The commercial Zn foil, a Pt plate, and a saturated calomel electrode were used as the working electrode, counter electrode, and reference electrode, respectively. The electrodeposition process was conducted at the current density of 5 A cm^–2^ for 20 s (the sample denoted as 520 3D Zn), 40 s (540 3D Zn), and 60 s (560 3D Zn). The electrode was then rinsed with deionized water three times and dried at 65 °C overnight. For comparison, the commercial Zn foil was used without the electrodeposition process (denoted as bare Zn).

### Theoretical Calculations

All the theoretical results in this work were calculated via plane-wave density functional theory using the Vienna Ab initio Simulation Package (VASP).^57–61^ The interaction between core and valence electrons was described using the projector augmented wave (PAW) method.^60^ We constructed a slab model using the Surfaxe package, with vacuum and slab thickness set to 30 Å. A 5 × 5 × 1 supercell for (001) and a 5 × 2 × 1 supercell for (101) of the slab model were employed to reduce finite size effects.^62^ All potential adsorption sites generated by the Pymatgen package were reduced to avoid symmetry equivalent positions.^63^ Four adsorption sites for the (001) plane and 10 adsorption sites for the (101) plane were identified and relaxed to their ground states using the PBEsol exchange-correlation functional.^64^ The energy and force convergence criteria were set to 10^–5^ eV and 10 meV Å^–1^, respectively. Geometry optimizations were performed using a plane-wave energy cutoff of 500 eV and Γ-centered 3 × 3 × 1 *k*-point mesh. The adsorption energy was calculated as

$$E_{\mathrm{ads}}=E_{\mathrm{tot}}-E_{\mathrm{slab}}-E_{\mathrm{Zn}}$$

where *E*_tot_ is the total energy of the slab with the adsorbed Zn, *E*_slab_ is the energy of the pristine slab, and *E*_Zn_ is the energy of an isolate Zn atom in a cubic unit cell of dimension 10 Å to avoid periodic boundary conditions.

### Electrodeposition of the PANI Electrode

The electrodeposition of PANI was conducted with a three-electrode system. The platinum wire and Ag/AgCl electrode were used as the counter and reference electrodes, respectively. A 2 × 2 cm graphene paper was first cleaned by UV-ozone treatment for 1 h and then directly used as the working electrode. 5.28 g of H_2_SO_4_ and 2.33 g of aniline were dissolved in 50 mL of distilled water as the electrodeposition electrolyte. The constant voltage of direct current electrodeposition was controlled at 0.85 V for 40 s. After electrodeposition, the electrodes were immersed in distilled water for cleaning and dried at 65 °C overnight.

### Synthesis of V_2_O_5_ Nanowires

A 0.364 g portion of the commercial V_2_O_5_ powder was dissolved in 30 mL of DI water and stirred at room temperature for 30 min. Then, 5 mL of H_2_O_2_ (30% w/v) was added into the solution with stirring until a transparent orange solution was observed. The obtained solution was transferred to a Teflon-lined autoclave and maintained at 180 °C for 96 days. The products collected by centrifugal separation were washed with ethanol and DI water several times and finally dried at 80 °C under a vacuum overnight.

### Preparation of V_2_O_5_ Cathodes

The as-prepared V_2_O_5_ nanowires were gently ground in a mortar. The fined powder was then mixed well with Super P and a PVDF binder, at a mass ratio of 7:2:1, in NMP by vortex mixing processes at 2000 rpm for 2 min for three times (THINKY ARM-310CE mixer). The obtained slurry was casted on the graphene paper by the doctor blade method and then dried at 70 °C under vacuum overnight.

### Preparation of AC Cathodes

The commercial AC paste was used directly as the active material. The ink was drop-casted on the carbon paper and subsequently heated at 65 °C overnight.

### Preparation of the Electrolytes

The 3 M Zn­(CF_3_SO_3_)_2_ aqueous electrolyte was prepared by dissolving 21.812 g of Zn­(CF_3_SO_3_)_2_ in 20 mL of DI water. This solution was continuously stirred overnight to form a uniform electrolyte. The 1 M Zn­(CF_3_SO_3_)_2_ aqueous electrolyte was prepared in the same procedure except adding 7.27 g of Zn­(CF_3_SO_3_)_2_ during the dissolving step.

### Characterizations

The crystal structures of the samples were examined by X-ray diffraction (XRD) analysis using a Malvern Panalytical Aeris with Cu Kα radiation. Raman spectra of the samples were acquired on a Renishaw inVia confocal Raman microscope with a 532 nm wavelength laser. The hydrophilicity of the samples was characterized by contact angle measurement (Attension Optical Tensiometer, Biolin Scientific). The in situ optical images were captured by a Zeiss Axio Scope.A1 Trinocular Pathology microscope. Acrylic molds with an H-shaped groove (length: 1 cm; width: 0.5 cm; depth: 0.5 cm) in the middle were utilized to simplify the in situ image capture process. The electrodes were positioned on both sides of the H-shaped curve, and then 3 M Zn­(CF_3_SO_3_)_2_ aqueous electrolyte was added to fill the pattern. Scanning electron microscopy (SEM) was conducted using ZEISS EVO LS15 to capture the morphology of the samples.

### The Fabrication of Symmetric Cells and Full Cells

Both full and half cells were constructed following an identical procedure. The glass microfiber filter paper (Whatman) was employed as the separator. CR2045 was selected as the battery component, and 3 M Zn­(CF_3_SO_3_)_2_ aqueous electrolyte was used for half cells and Zn//PANI and Zn//V_2_O_5_ full cells. In half cells, the same kind of Zn electrodes were used on both sides. In Zn//PANI and Zn//V_2_O_5_ full cells, the prepared Zn electrode was the anode while PANI or V_2_O_5_ on graphene paper was used as the cathode. Zn//AC ZICs were assembled with the same procedure except using CR2032 as the battery component and 1 M Zn­(CF_3_SO_3_)_2_ aqueous electrolyte.

### Symmetric Cell Tests

The galvanostatic discharge/charge (GDC) profile was obtained by Neware battery testers at different areal currents, including 0.1, 0.5, 1, 2, and 5 mA cm^–2^. The long-term cycling tests was measured by Neware battery testers with an areal current of 1 mA and an areal capacity of 1 mAh cm^–2^. The linear sweep voltammetry (LSV) tests and the Tafel plots were conducted at a scan rate of 50 mV s^–1^ by a VIONIC electrochemical workstation with a three-electrode system. The prepared Zn electrode served as the working electrode, Pt wire as the counter electrode, and Ag/AgCl electrode as the reference electrode. The electrochemical impedance spectroscopy (EIS) test was completed by Alvatek V83192 at various temperatures, including 25, 30, 35, 40, 45, 50, 55, 60, and 65 °C, within a frequency range from 0.01 to 10^6^ Hz. The chronoamperometry (CA) test was carried out with a constant overpotential of 150 mV for 600 s in symmetrical Zn/Zn cell configuration (Alvatek V83192). The Zn^2+^ transference number (*t*_Zn_) was obtained by another CA test with a constant overpotential of 10 mV and EIS before and after the CA test.

### Full Cell Tests

Both half and full cells were tested on the same instruments. The cyclic voltammetry (CV) profiles of Zn//PANI and Zn//AC were acquired on Alvatek R54390 with scan rates from 0.6 to 1.5 mV s^–1^ and from 2 to 50 mV s^–1^, respectively. The galvanostatic discharge/charge (GDC) tests of Zn//PANI and Zn//AC were measured by Neware battery testers with specific currents from 100 to 2000 mA g^–1^ and from 200 to 2000 mA g^–1^, respectively. The long-term cycling performances of Zn//PANI, Zn//V_2_O_5_, and Zn//AC were all measured by Neware battery testers at 1, 1, and 5 A g^–1^, respectively. The electrochemical impedance spectroscopy (EIS) tests for all the full cells were completed within a frequency range of 0.01–10^6^ Hz (Alvatek V83192). Zn//PANI, Zn//V_2_O_5_, and Zn//AC full cells were tested within the voltage window from 0.5 to 1.5 V, 0.2 to 1.6 V, and 0.2 to 1.8 V, respectively.

## Supplementary Material



## Notes

Cross-section SEM images (Figure S1); calculation of intensity ratio of *I*_(101)_/*I*_(002)_ (Table S1); adsorption energy calculations (Figures S2–S5); calculation total energy and adsorption energy (Table S2); long-term cycling tests (Figures S6 and S7); post-SEM images (Figure S8); Nyquist plots (Figure S9); activation energy calculations (Figure S10); comparative Zn^2+^ transference number (Figure S11); in situ optical images (Figure S12); post-SEM image (Figure S13); in situ optical images (Figure S14); post-SEM images (Figure S15); in situ photo images of the symmetric cells (Figure S16); post-cross-sectional SEM images (Figure S17); post-SEM images (Figure S18); SEM images and Raman of PANI, V_2_O_5_, and AC cathodes (Figures S19 and S20); CV curves of capacitors (Figure S21); GDC curves of the Zn//PANI full cell (Figure S22); GDC curves of capacitor (Figure S23); Raman spectra and SEM images of PANI cathodes after cycling (Figures S24 and S25); SEM images of the Zn anodes after cycling (Figure S26); Nyquist plots (Figure S27); equivalent circuit model (Figure S28); and simulated values (Table S3) (PDF)
