# Supplementary material for: 3D Porous Zinc Scaffold Anodes for Enhanced Stability and Performance in Zinc-Ion Energy Storage Systems
Source: ACS Nano. 2025 Jul 8;19(28):26147–60. doi: 10.1021/acsnano.5c07729 (PMC12291595; doi:10.1021/acsnano.5c07729)
Supplement: Supplementary file 1 [file nn5c07729_si_001.pdf]

Supporting Information

For

3D Porous Zinc Scaffold Anodes for Enhanced Stability and Performance in Zinc-ion Energy Storage Systems

Xiaopeng Liu,[1] Ruiqi Wu,[2] Xueqing Hu,[1] Alex M. Ganose,[2] Jingli Luo,[1] Iman Pinnock,[1] Nibagani Naresh,[1] Yijia Zhu,[1] Yujia Fan,[1] Tianlei Wang,[3] Shuhui Li,[3] Ivan P. Parkin,[3] Buddha Deka Boruah*,[1]

[1]Institute for Materials Discovery, University College London (UCL), London, WC1E 7JE, UK

[2]Department of Chemistry, Imperial College London (ICL), London, W120BZ, UK

[3]Department of Chemistry, University College London (UCL), London, WC1H 0AJ, UK

**Corresponding Author:*

Dr. Buddha Deka Boruah, E-mail: b.boruah@ucl.ac.uk

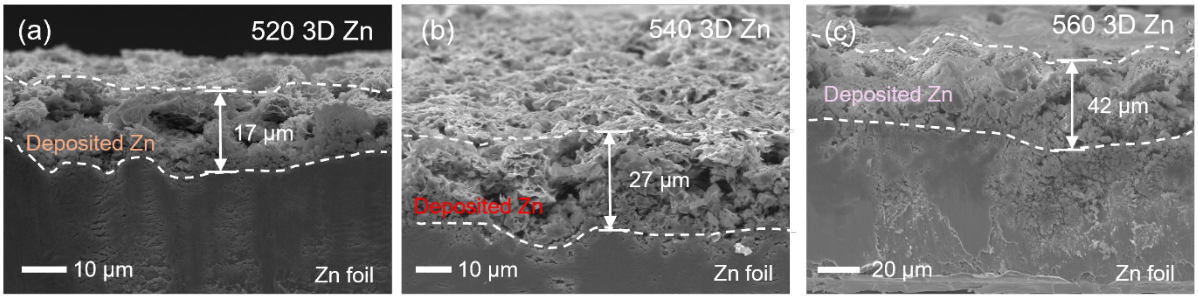

Figure S1. *Cross-section SEM images of (a) 520, (b) 540 and (c) 560 3D Zn.*

Table S1. *the calculated intensity ratio of $I_{(101)}/I_{(002)}$ for the prepared Zn electrodes.*

Sample Name	$I_{(101)}/I_{(002)}$
Bare Zn	1.13
520 3D Zn	1.71
540 3D Zn	1.78
560 3D Zn	1.16

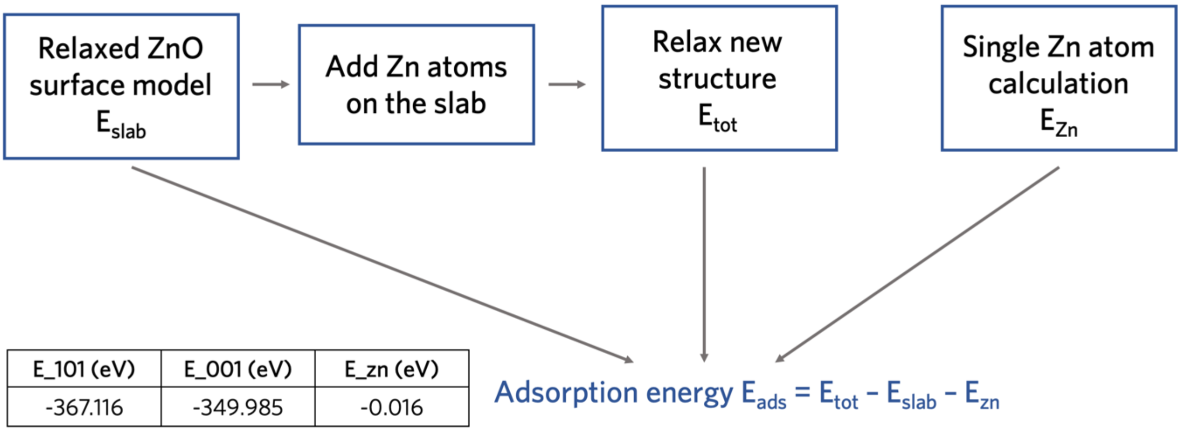

Figure S2. *Workflow to calculate the adsorption energy of Zn single atom on the Zn surface.*

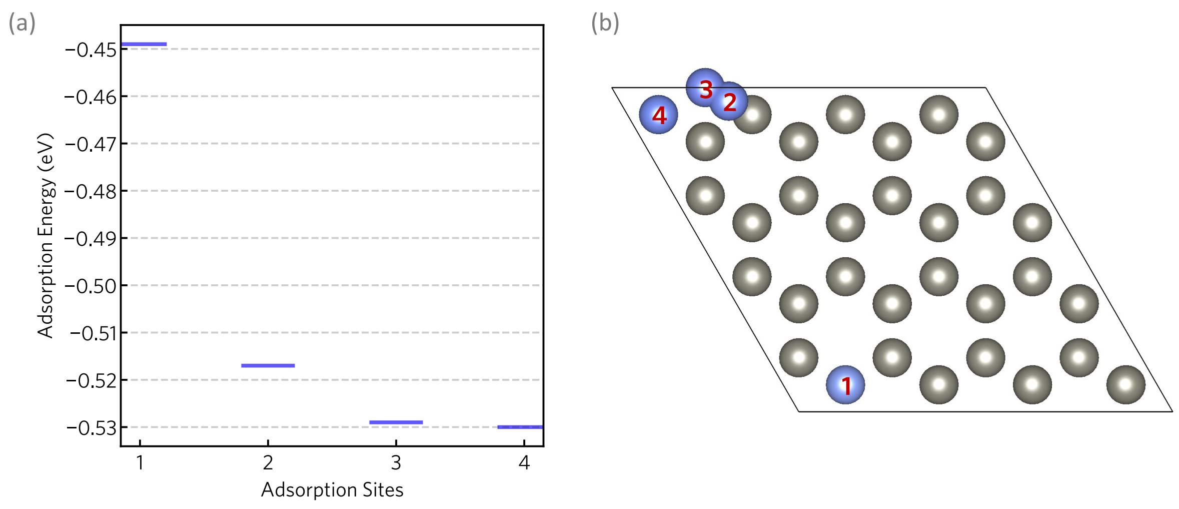

Figure S3. *Calculated adsorption energy and adsorption sites for plane (001)*

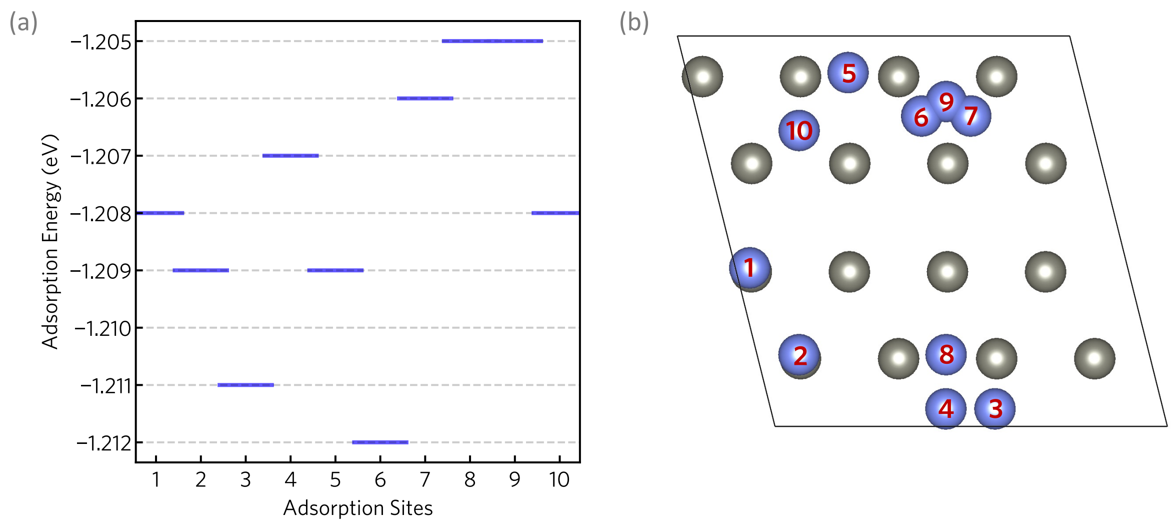

Figure S4. *Calculated adsorption energy and adsorption sites for plane (101)*

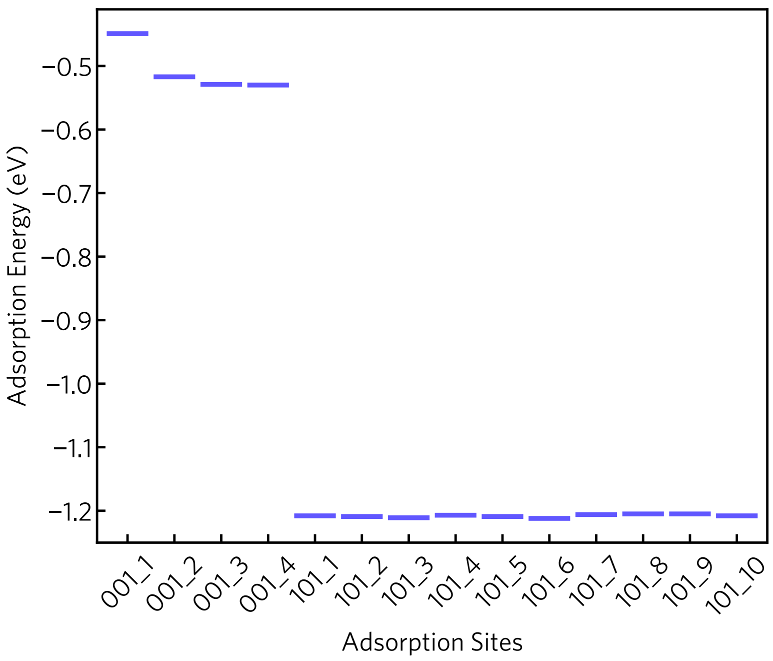

Figure S5. *Calculated adsorption energy for all the potential adsorption sites*

Table S2. *Calculated total energy and adsorption energy for different adsorption sites on (001) and (101) Zn surfaces.*

	Adsorption site	E (eV)	E_{ads} (eV)
001	0	-350.450	-0.449
	1	-350.518	-0.517
	2	-350.530	-0.529
	3	-350.531	-0.530
101	0	-368.340	-1.208
	1	-368.341	-1.209
	2	-368.343	-1.211
	3	-368.339	-1.207
	4	-368.341	-1.209
	5	-368.344	-1.212
	6	-368.338	-1.206
	7	-368.337	-1.205
	8	-368.337	-1.205
	9	-368.340	-1.208

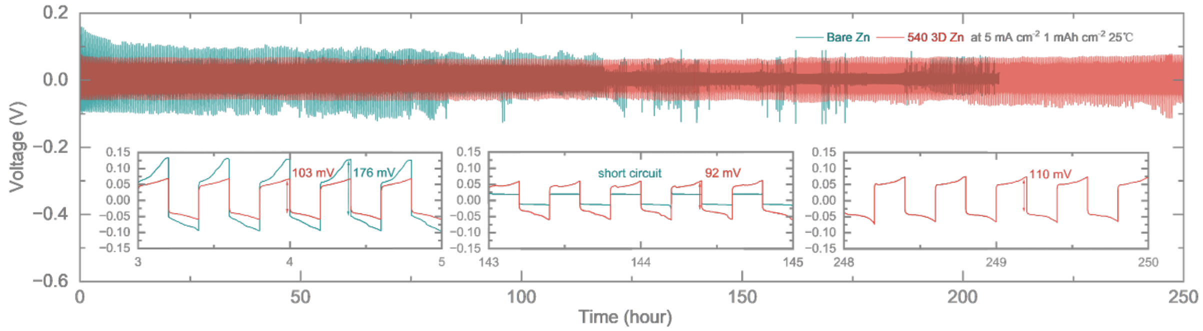

Figure S6. *Long-term cycling tests of the symmetric cells at an areal current of 5 mA cm^{-2}.*

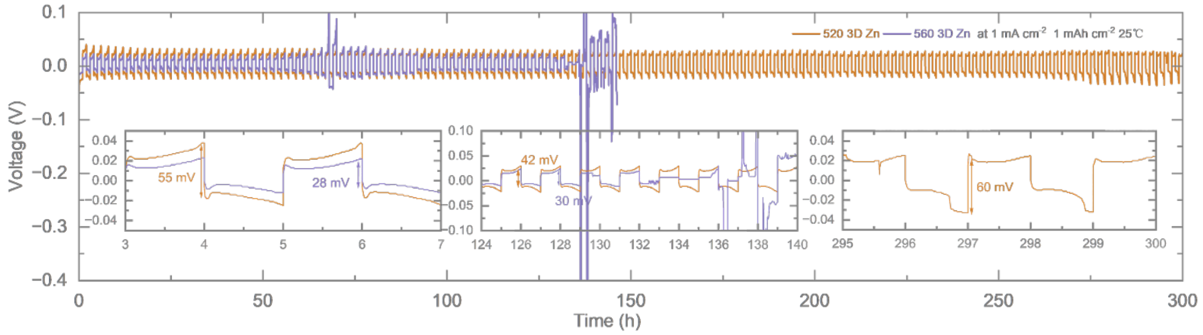

Figure S7. *Long-term cycling tests of the symmetric cells at an areal current of 1 mA cm^{-2}.*

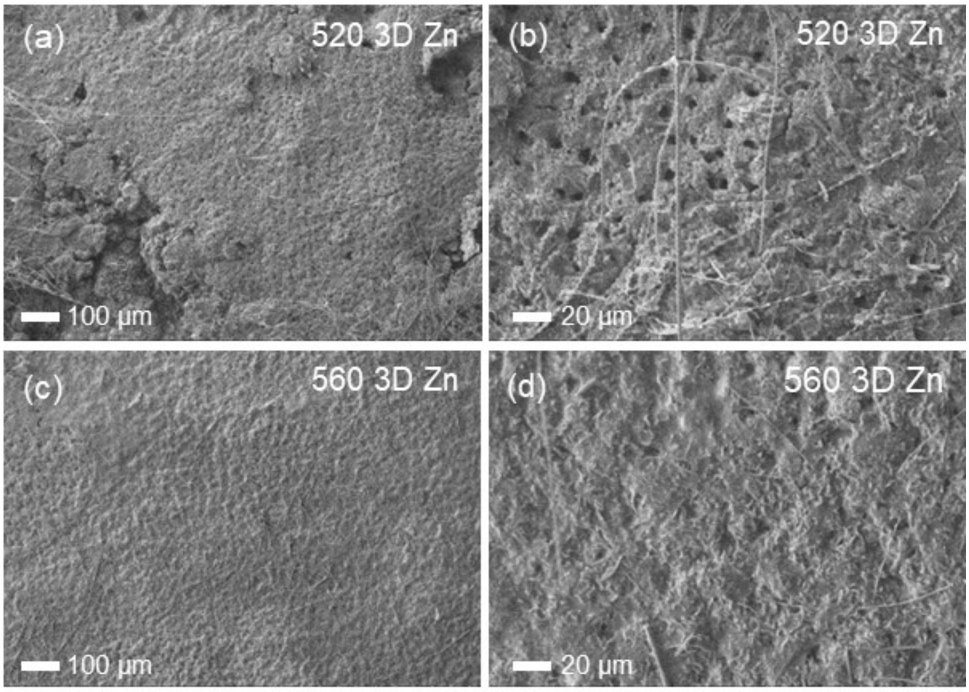

Figure S8. *Post-SEM images of the cycled (a, b) 520 3D Zn and (c, d) 560 3D Zn electrodes at different magnifications.*

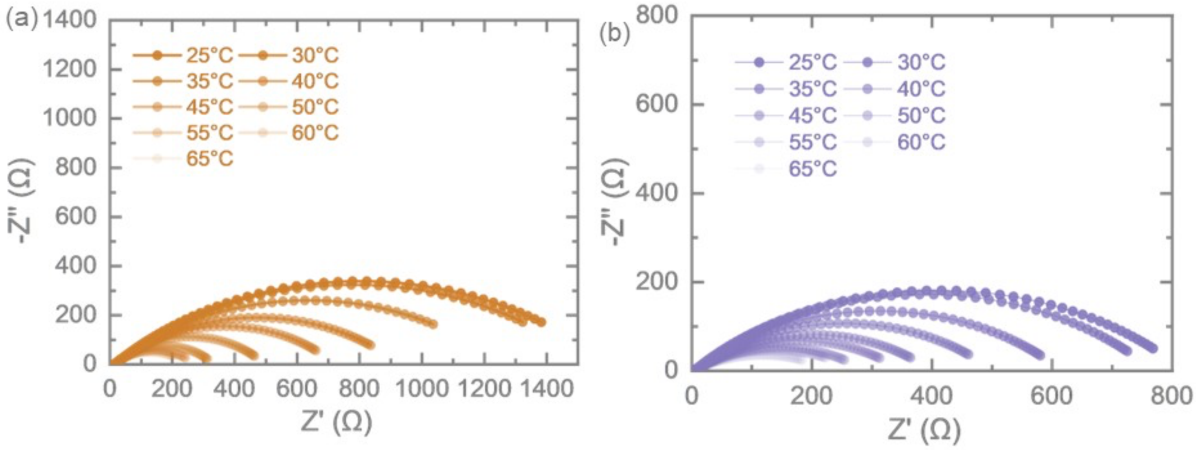

Figure S9. *Nyquist plots of the symmetric cells based on (a) 520 3D Zn and (b) 560 3D Zn at different temperature.*

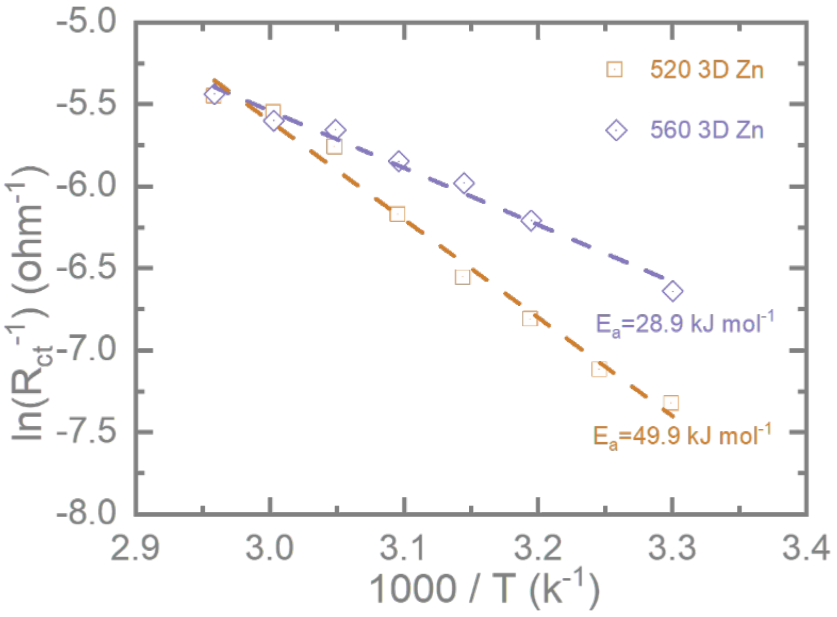

Figure S10. *Comparative activation energy plot for 520 3D Zn and 560 3D Zn anodes.*

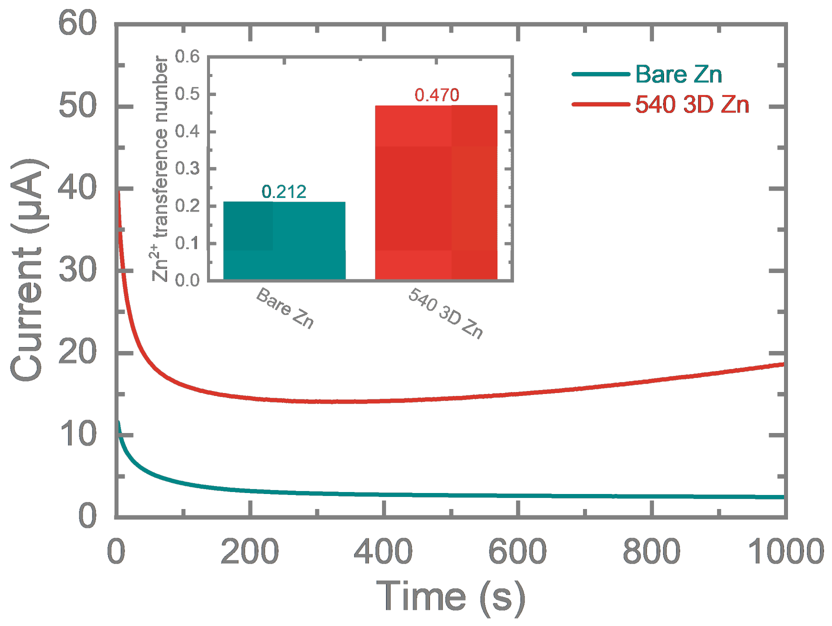

Figure S11. *Comparative* Zn^{2+} *transference number for bare Zn and 540 3D Zn.*

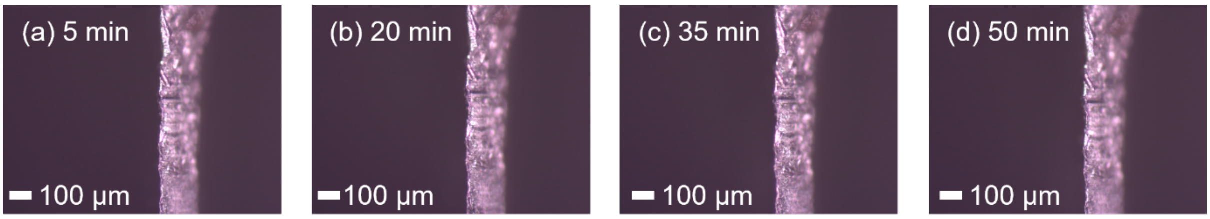

Figure S12. *In situ optical images for the Zn deposition process of 540 3D Zn captured at different time intervals.*

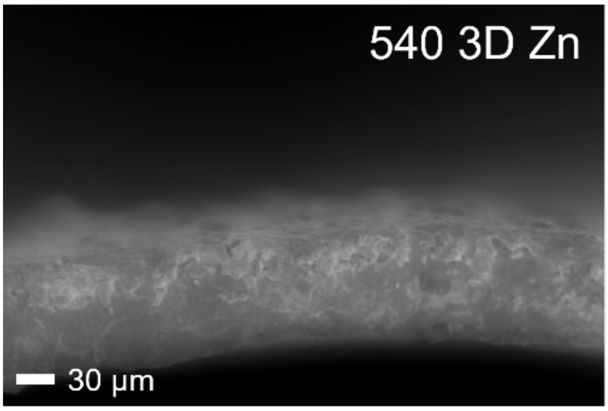

Figure S13. *Post-SEM image of the 3D 540 Zn from cross-section view after 60 min Zn^{2+} plating.*

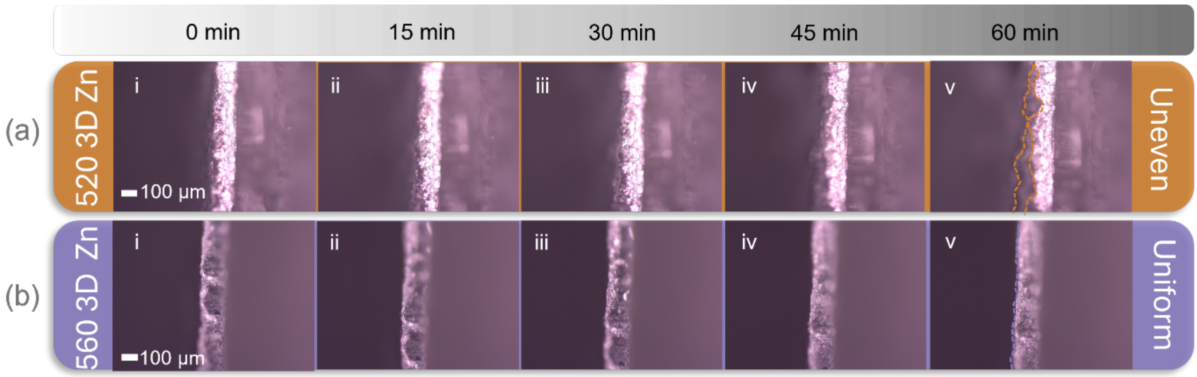

Figure S14. *In situ optical images for the Zn deposition process of (a) 520 and (b) 540 3D Zn captured at different time intervals.*

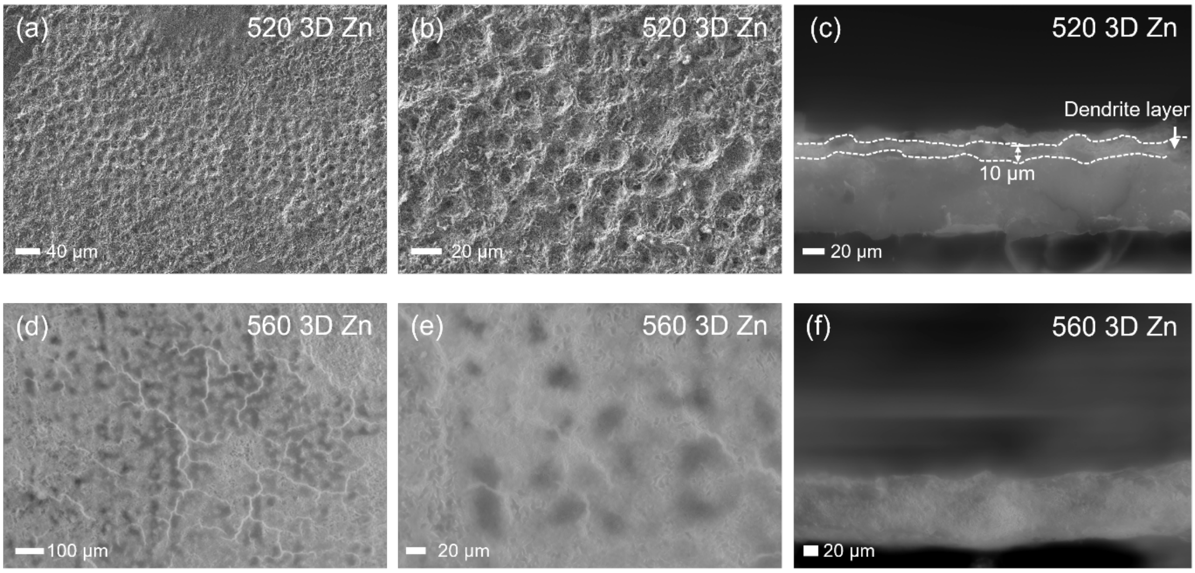

Figure S15. *Post-SEM images of the 520 3D Zn (a,b) from top view and (c) cross-section view after 60 min Zn^{2+} plating. Post-SEM images of the 560 3D Zn (d,e) from top view and (f) cross-section view after 60 min Zn^{2+} plating.*

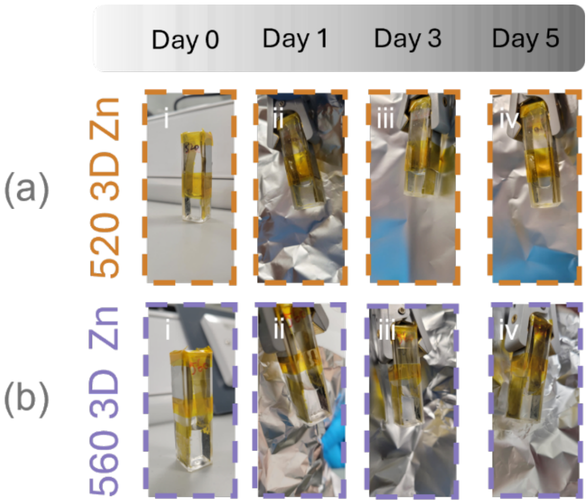

Figure S16. *In situ photo images of the symmetric cells with (a) 520 3D Zn and (b) 560 3D Zn in a 1 × 1 cm^2 cuvette cell for 5 days to observe Zn plating/stripping behaviour.*

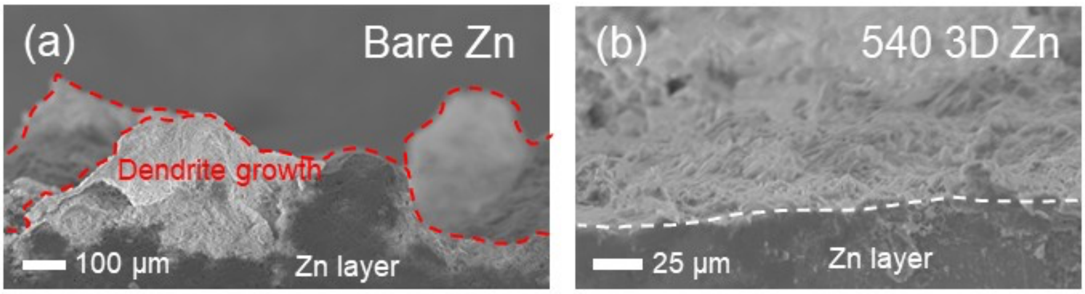

Figure S17. *Post cross-sectional SEM images of (a) bare Zn and (b) 540 3D Zn after cycling in the cuvette cell for 5 days.*

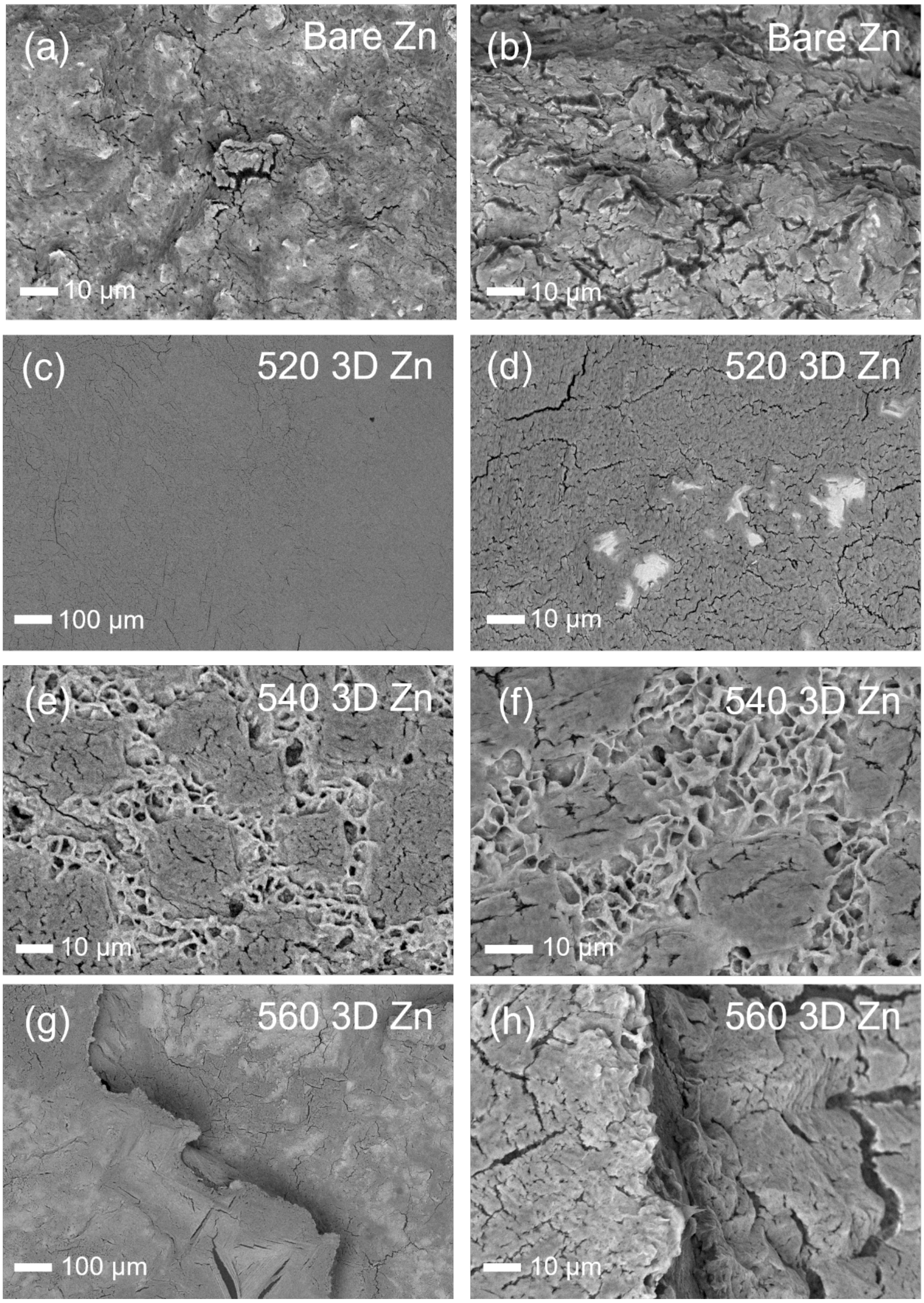

Figure S18. *Post-SEM images of (a,b) bare Zn, (c,d) 520 3D Zn, (e,f) 540 3D Zn and (g,h) 560 3D Zn after cycling in the cuvette cell for 5 days.*

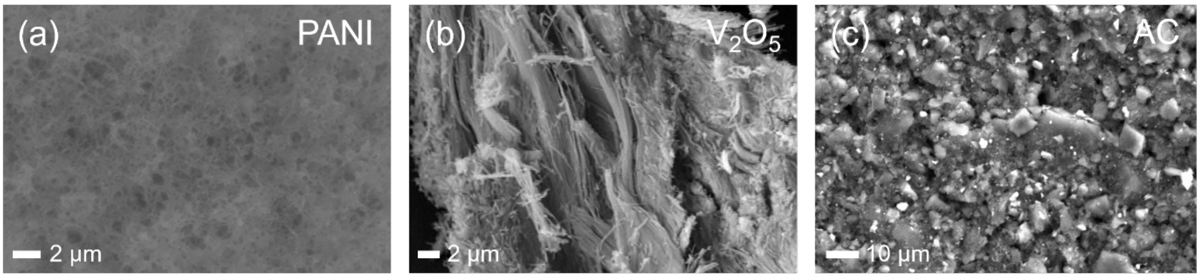

Figure S19. *SEM images of (a) PANI, (b)V_2O_5 and (c) AC cathodes.*

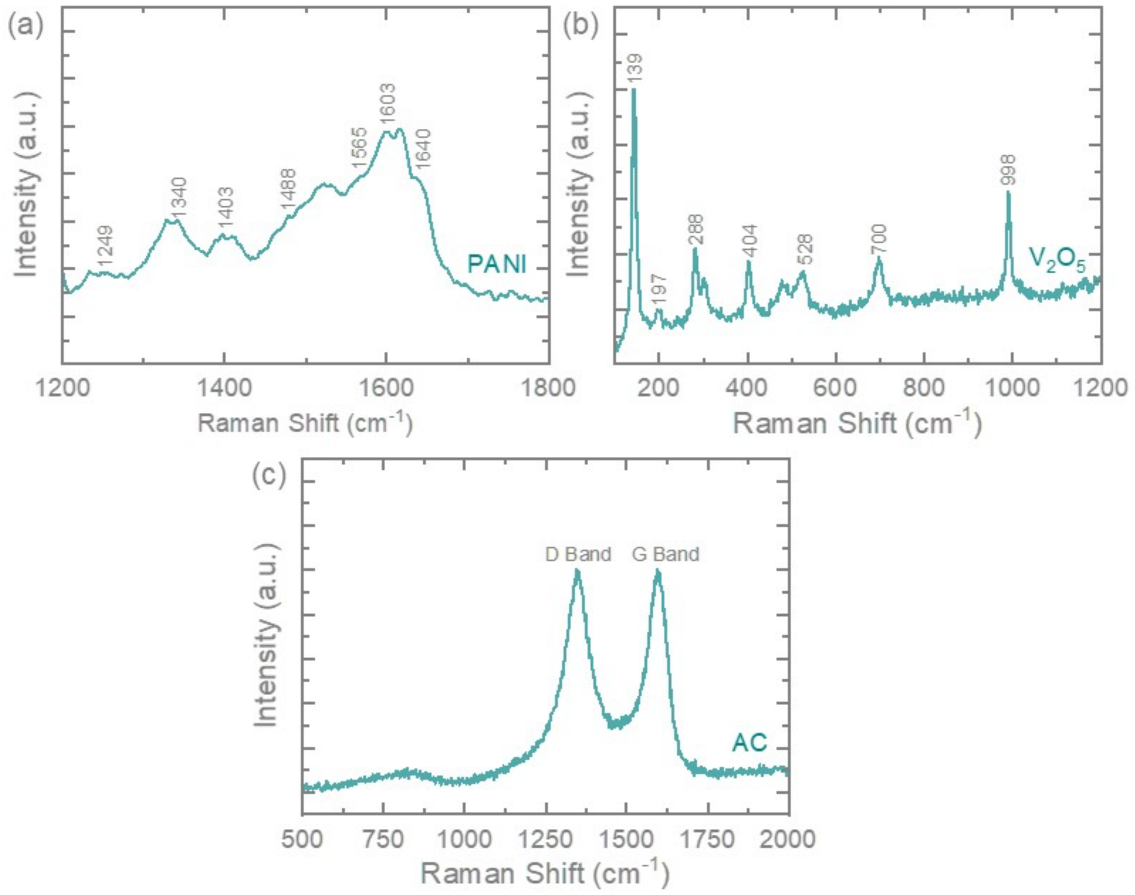

Figure S20. *Raman spectra of (a) PANI, (B) V_2O_5 and (c) AC cathodes.*

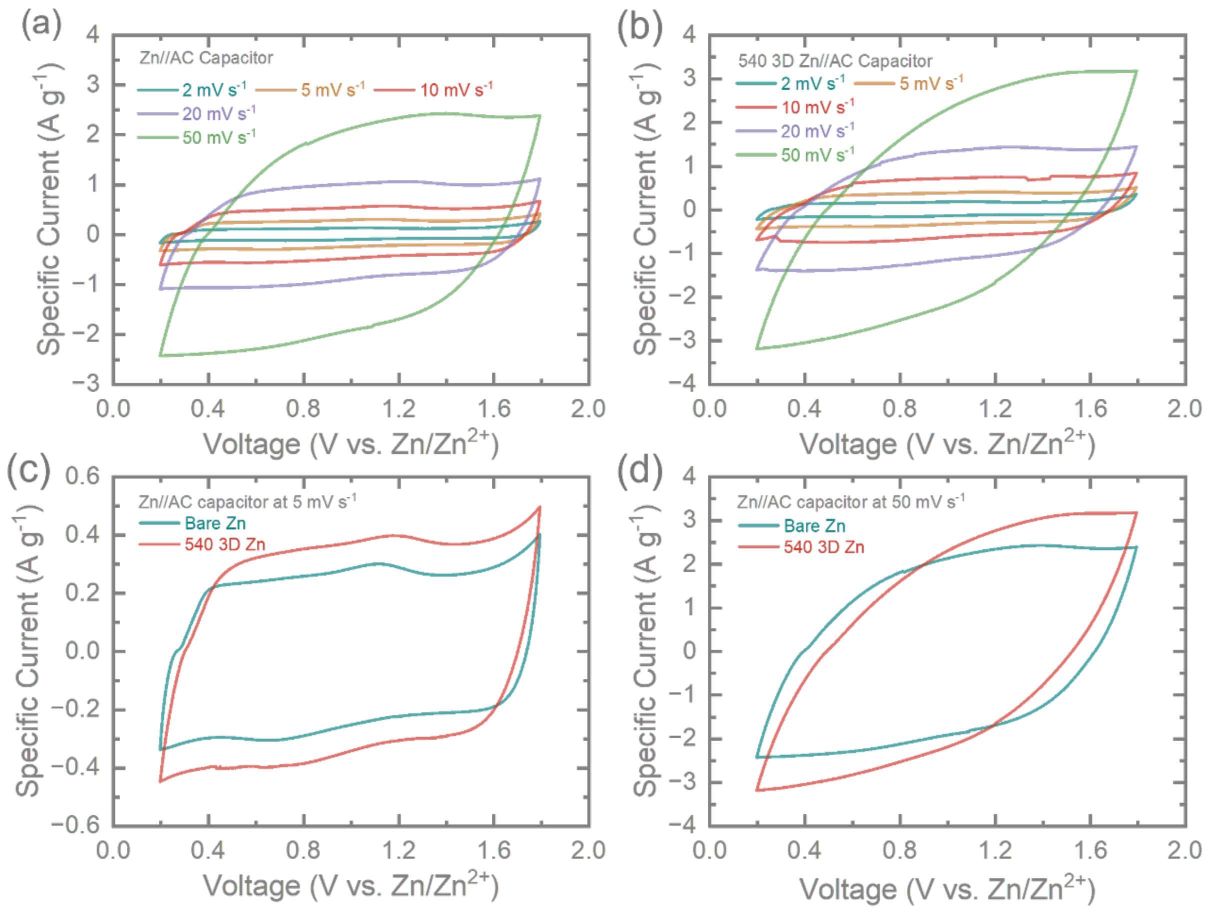

Figure S21. *CV curves of (a) bare Zn//AC and (b) 540 3D Zn//AC capacitors. Comparative CV curves of the prepared ZICs at different scan rates of (c) 5 mV* s^{-1}*, (d) 50 mV* s^{-1}*.*

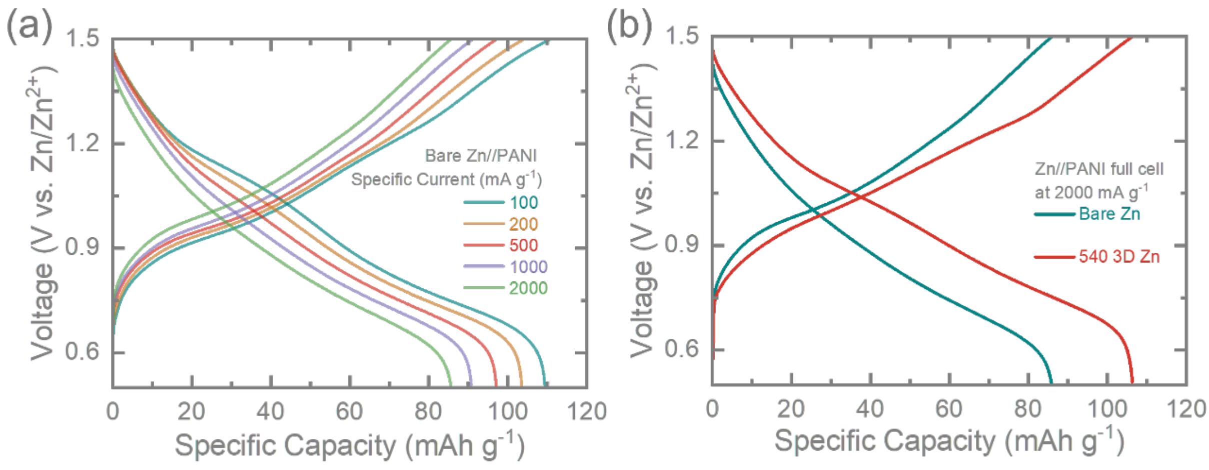

Figure S22. *(a) GDC curves of Zn//PANI full cell at different current densities. (b) Comparative GCDs of Zn//PANI and 540 3D Zn//PANI full cells at specific currents of 2,000 mA* g^{-1}*.*

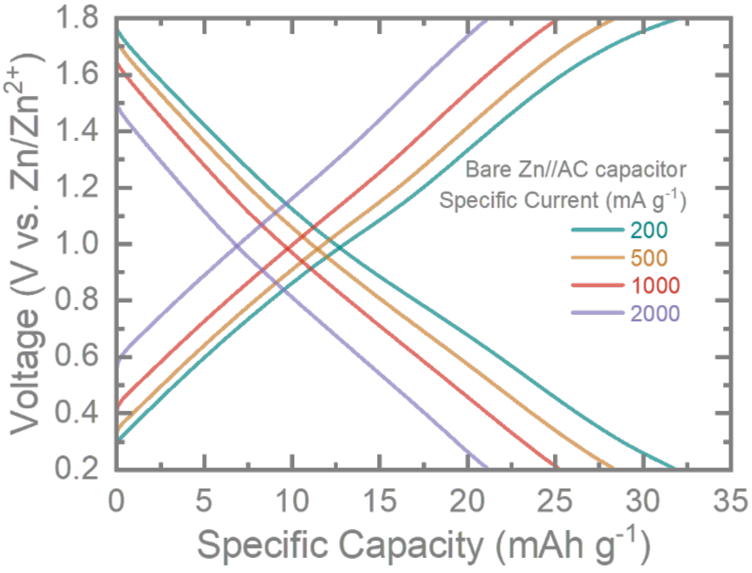

Figure S23. *(a) GDC curves of Zn//AC capacitor at different specific currents.*

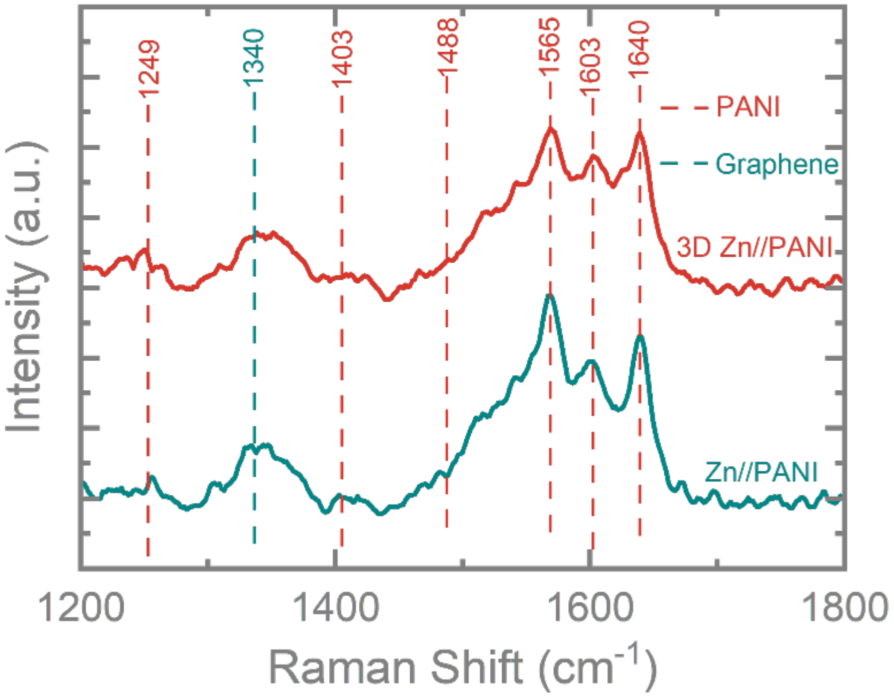

Figure S24. *Raman spectra of PANI cathodes in Zn//PANI and 540 3D Zn//PANI after cycling.*

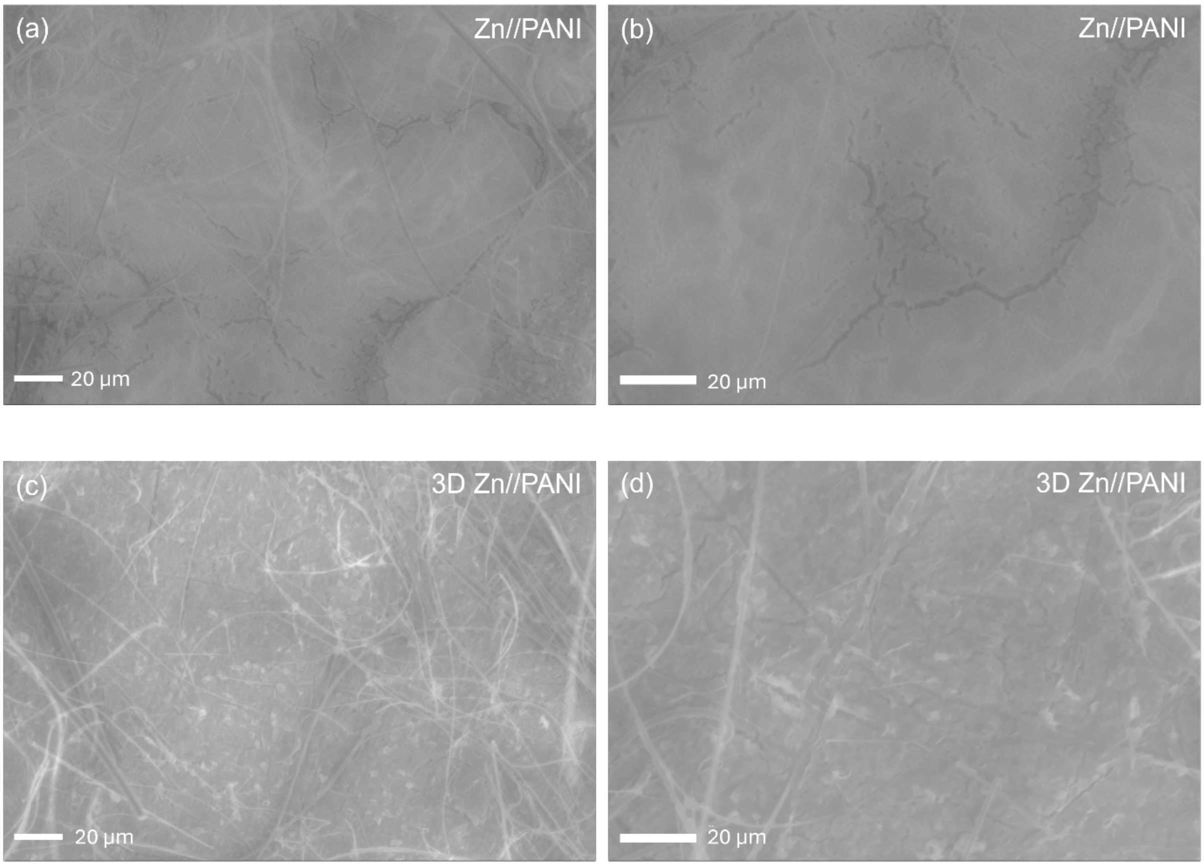

Figure S25. *SEM images of the PANI in (a,b) Zn//PANI and (c,d) 540 3D Zn//PANI after cycling.*

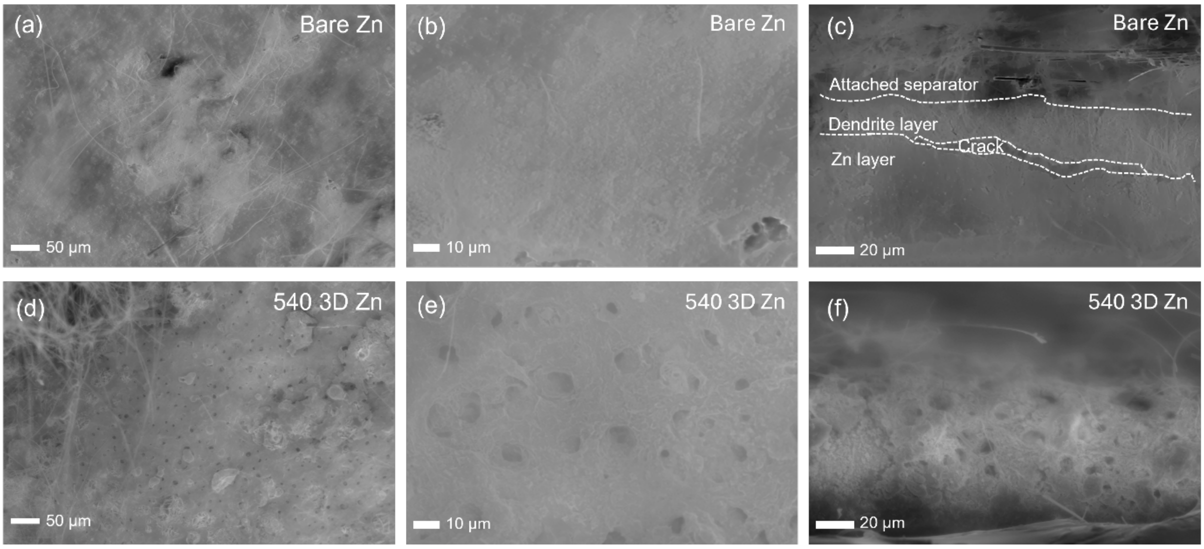

Figure S26. *SEM images of the Zn anode from Zn//PANI at (a,b) top view and (c) cross-section view, and from 540 3D Zn//PANI (d,e) top view and (f) cross-section view after cycling.*

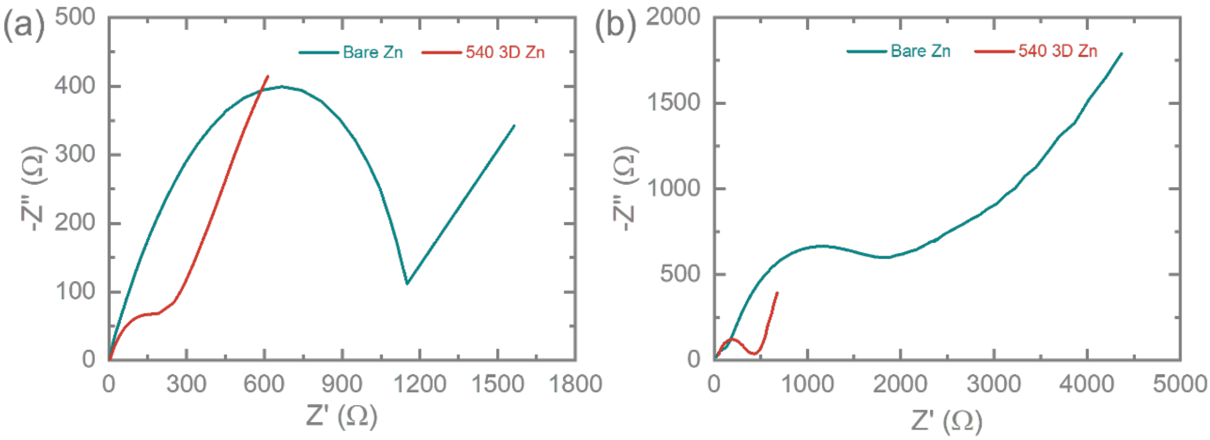

Figure S27. *Comparative Nyquist plots of Zn//PANI and 540 3D Zn//PANI (a) before cycling and (b) after cycling.*

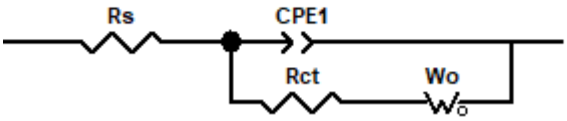

Figure S28. *Representation of the equivalent circuit model to interpret the EIS results for Figure S27.*

Table S3. *The simulated values of Electrochemical Impedance Spectroscopy in Figure S27.*

	Rs (Ω)	Rct (Ω)
Bare Zn//PANI (Before)	13	1309
Bare Zn//PANI (After)	89	2405
540 3D Zn//PANI (Before)	3	272
540 3D Zn//PANI (After)	23	389
